# Protein kinase A controls yeast growth in visible light

**DOI:** 10.1186/s12915-020-00867-4

**Published:** 2020-11-16

**Authors:** Mikael Molin, Katarina Logg, Kristofer Bodvard, Ken Peeters, Annabelle Forsmark, Friederike Roger, Anna Jörhov, Neha Mishra, Jean-Marc Billod, Sabiha Amir, Mikael Andersson, Leif A. Eriksson, Jonas Warringer, Mikael Käll, Anders Blomberg

**Affiliations:** 1 Department of Chemistry and Molecular Biology, University of Gothenburg, Gothenburg, Sweden; 2 Department of Biology and Biological Engineering, Chalmers University of Technology, Gothenburg, Sweden; 3 Department of Physics, Chalmers University of Technology, Gothenburg, Sweden; 4 Horizon Discovery, Cambridge, CB25 9TL UK; 5 Bio-Me A/S, Oslo Science Park, Gaustadalléen, 210349 Oslo, Norway

**Keywords:** Visible light, Light sensitivity, Genome-wide screen, Yeast, Protein kinase A, HOG signaling, Diphthamide modification

## Abstract

**Background:**

A wide variety of photosynthetic and non-photosynthetic species sense and respond to light, having developed protective mechanisms to adapt to damaging effects on DNA and proteins. While the biology of UV light-induced damage has been well studied, cellular responses to stress from visible light (400–700 nm) remain poorly understood despite being a regular part of the life cycle of many organisms. Here, we developed a high-throughput method for measuring growth under visible light stress and used it to screen for light sensitivity in the yeast gene deletion collection.

**Results:**

We found genes involved in HOG pathway signaling, RNA polymerase II transcription, translation, diphthamide modifications of the translational elongation factor eEF2, and the oxidative stress response to be required for light resistance. Reduced nuclear localization of the transcription factor Msn2 and lower glycogen accumulation indicated higher protein kinase A (cAMP-dependent protein kinase, PKA) activity in many light-sensitive gene deletion strains. We therefore used an ectopic fluorescent PKA reporter and mutants with constitutively altered PKA activity to show that repression of PKA is essential for resistance to visible light.

**Conclusion:**

We conclude that yeast photobiology is multifaceted and that protein kinase A plays a key role in the ability of cells to grow upon visible light exposure. We propose that visible light impacts on the biology and evolution of many non-photosynthetic organisms and have practical implications for how organisms are studied in the laboratory, with or without illumination.

## Background

Light is a double-edged sword: it is essential for life on the planet but also causes cell damage and death. Consequently, organisms have evolved systems not only for harvesting and converting light energy into chemical energy, but also for resisting its toxic effects. The photoprotective role of plastoquinone-9 [1] and the dissipation of absorbed light as heat involving xanthophyll [2] are examples.

A broad variety of photosynthetic and non-photosynthetic organisms, like filamentous fungi, sense, and respond to light [3]. Adaptation to DNA and protein damage caused by ultraviolet (UV, < 400 nm) wavelengths [3, 4] may explain the wide distribution of some of the light protection systems across the tree of life. UVA causes single-strand breaks in DNA, while UVB stimulates formation of thymine and cytosine dimers and causes double-strand DNA breakage. Protecting DNA from these mutagenic effects of high-energy UV light is essential, as illustrated by the co-evolution of the light-sensory and DNA repair roles of photolyases [5]. In proteins, the indole group of tryptophan is the dominant UV absorber [6, 7]. Indole excitation damages protein structure and integrity and may convert proteins into photosensitizers that produce reactive oxygen species (ROS). ROS in turn reacts intracellularly with many biomolecules, including DNA, causing cellular dysfunction and mutation [8]. Many organisms have adapted to avoid the toxic effects of UV absorption by protein and DNA by evolving constitutive or UV-induced production of pigments, such as carotenoids or melanins, which are broadband UV and visible light absorbers that are capable of nonradiative dissipation of up to 99.9% of the absorbed light [9]. In these organisms, defective pigment biosynthesis genes consequently confer high UV sensitivity [10].

The photobiology of visible light (400–700 nm) is much less understood than that of UV light. The toxicity from visible light is usually ascribed to the light-induced production of ROS by endogenous photosensitizers, such as flavins and porphyrins [11, 12]. Fungi sense visible light through the flavin-binding photoreceptor White Collar 1 (WC-1) which together with its interaction partner WC-2 acts as a light-activated transcription factor [3]. Opsins, phytochromes, and cryptochromes also have photosensory roles in some fungi but are absent in the key model yeast *Saccharomyces cerevisiae* [13]. *S*. *cerevisiae* instead senses light through a peroxisomal, flavin-containing acyl-CoA oxidase, Pox1, which releases H_2_O_2_ and initiates a H_2_O_2_-induced signaling cascade [14]. Other flavin-associated proteins may also be excited by visible light and could contribute to either light sensing and signaling or light toxicity, the latter through photosensitization and ROS production [11, 12]. Only the succinate dehydrogenase, Sdh1, and the L-arabinono-1,4-lactone oxidase, Alo1, covalently bind flavin in yeast. Still, 1% of yeast proteins, and 1–3% of bacterial and eukaryotic proteins overall, transiently bind to the flavin-containing metabolites FMN (flavin mononucleotide) or FAD (flavin adenine dinucleotide) [15, 16] and use these as co-factors to drive chemical reactions.

The inner mitochondrial membrane is particularly rich in flavin and porphyrin binding proteins, in the form of blue light absorbing cytochromes. Blue light absorption has been reported to damage cytochromes in an oxygen dependent manner, ending mitochondrial respiration [17]. Furthermore, the absence of mitochondrial DNA, and of cytochromes, confers light resistance [18]. Visible light also controls the oscillatory metabolic switching between fermentation and respiration in starving yeast cells with the strongest effects conferred by wavelengths matching the absorbance maxima of cytochromes [19]. Ultimately, visible light damages DNA [20], disturbs mitochondria [21], suppresses mitosis [22], and inhibits respiration, protein synthesis, and membrane transport [18]. But whether these effects are directly or indirectly due to light and what the underlying mechanisms are is not known. That visible light activates the H_2_O_2_-induced transcription factor Yap1 and that cells are hyper-sensitive to visible light in the absence of Yap1 supports a model where light toxicity depends on ROS generation [19].

We exposed the *S*. *cerevisiae* gene knockout collection to visible light at moderately stressful intensities comparable to those that yeast often experiences in nature and measured growth defects. We found HOG pathway signaling, transcription, protein translation, and the oxidative stress response to stand out as required for normal visible light resistance. Most of the mutants sensitive to light showed reduced shuttling of the transcription factor Msn2 to the nucleus and decreased glycogen accumulation, both markers of an abnormally high activity of protein kinase A (cAMP-dependent protein kinase, PKA). We therefore used a fluorescent PKA reporter and mutants genetically engineered to have constitutively high or low PKA activity to show that PKA repression is required for light resistance and thus appears to be a common denominator.

## Results

### Developing a screen for genes required for growth in visible light

**Fig. 1:**
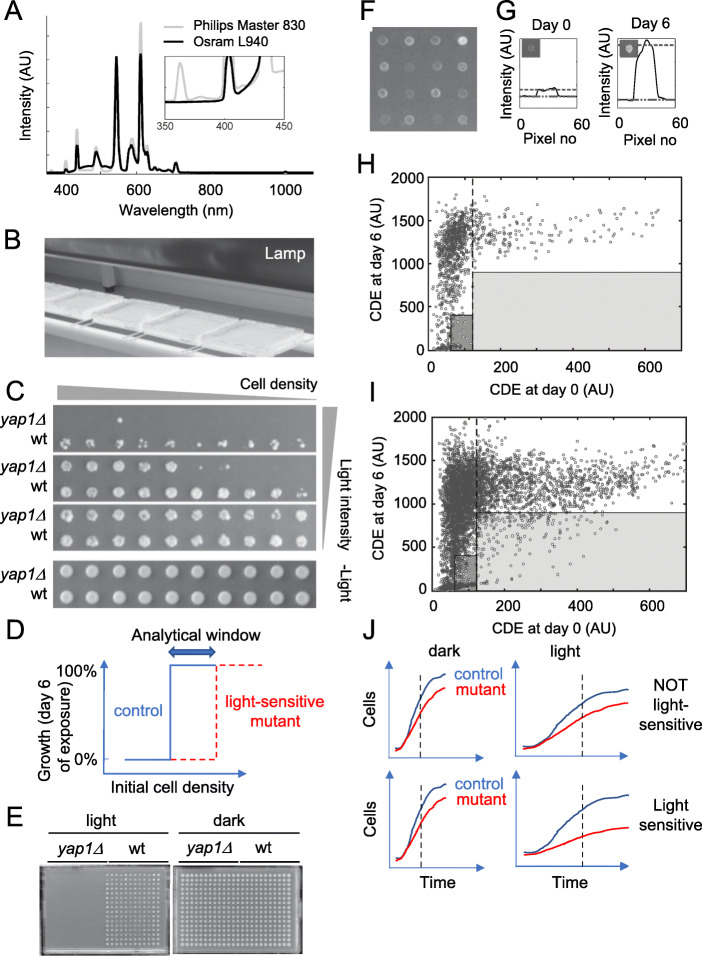
Development and optimization of the pipeline for genome-wide screening for light-sensitive mutants. **a** The spectrum of the UV-filtered fluorescent lamp, Osram L 940 with the warm white color code 830, in comparison to the spectrum of a normal fluorescent lamp in the lab. Inset shows an enlargement of the 350–450 nm range. Note that the peak in the UV range at ~ 360 nm in the normal lamp is absent for the UV filtered Osram L 940. **b** Experimental design of the set-up for the genome-wide screen of the light sensitivity of the deletion mutants. The agar plates are placed on wooden sticks to maximize air-flow and cooling. The lamp could be adjusted to various heights to optimize the scoring of light sensitivity. **c** Both light intensity and the initial cell density affect the light sensitivity of yeast strains, seen on wild type and *yap1∆* mutant strains. **d** Schematic drawing that exemplifies the methodological challenge, with all strains being light sensitive at lower initial cell densities, and none at high initial cell densities. In between, there is an analytical window which is to be used for scoring light-sensitive mutants. **e** Images show the contrast in growth between the wild type and the positive control *yap1∆* using the same procedure and format (384 colonies/plate) as in the genome-wide screening experiments. **f** Example of the variation in cell density at day 0 directly after pinning. **g** Image analysis procedure for the estimation of light sensitivity of the deletion mutants. Illustration of how the cell density was estimated by measuring the intensity over each colony in the image. Both a background intensity value and a colony intensity value were extracted and the difference in intensity was taken as the cell density estimate (CDE). **h** Experimental distribution of the 1064 control strains present at 76 copies per plate. An asymmetrical distribution of the control cells is clearly apparent, with colonies with a CDE at day 0 ≥ 121 (dotted line) showing 100% growth response during light exposure (day 6 data). At a CDE day 0 < 121, the light sensitivities of the control strains are clearly much more variable. High confidence set (light shaded box) and moderate confidence set (dark shaded box) are indicated (scoring criterium I). **i** The light sensitivity distribution of the mutants. High confidence set (light shaded box) and moderate confidence set (dark shaded box). **j** Schematic growth curves of light-sensitive and not light-sensitive mutants according to the scoring procedure used taking both slow growth in light as well as in the dark into account (scoring criterium II)

We first established a platform for surveying the growth of thousands of yeast strains under exposure to visible light. We arranged yeast colonies in dense, solid medium arrays and exposed them to visible light from an UV-filtered fluorescent lamp emitting light in evenly distributed intensity peaks between 400 and 700 nm (Fig. 1a). We positioned the lamp above the experimental plates, with good airflow around the plates to avoid confounding heat effects (Fig. 1b). The *yap1∆* mutant was used as a positive control since it is the only reported light-sensitive strain [19]. We first attempted to transfer cells to experimental plates from solid medium pre-cultures, but then found no light-induced growth defect for *yap1∆*, regardless of the distance to the lamp (data not shown). We hypothesized that this was due to the large number of cells (~ 100,000) transferred and by outer cells shielding inner cells in the nascent experimental colony by absorbing the light energy; the majority of cells would thereby avoid the toxic light effects and divide normally. We therefore instead transferred cells to agar from liquid pre-cultures to reduce the initial cell density and now registered a clear light-induced growth defect of cells lacking Yap1. This growth defect was stronger when fewer cells were transferred (Fig. 1c) and at very low initial cell densities the wildtype growth was impaired as well. We conclude that the visible light toxicity depends on the density of cells, likely because outer cells shield inner cells by absorbing the light. We found a sweet-spot cell density range—the analytical window—where the wild type/control growth was lightly, and the *yap1∆* growth heavily, light-dependent and at which a screen for genes required for normal growth in visible light could be performed (Fig. 1d).

We refined the pre-cultivation and transfer protocols to maximize the cell density homogeneity across cultures (see the “Methods” section). This allowed us to consistently distinguish wild type from *yap1∆* growth also in high throughput format (Fig. 1e). Finally, we developed an automated image analysis pipeline (Fig. 1f, g) that calculates a cell density estimate (CDE) based on the colony pixel intensity in photographs of the plates during illumination and in the dark (Additional file 1: Fig. S1). We used our high throughput set-up to screen the haploid mutant library, containing 4697 single gene deletion mutants. We used 1066 genetically identical control colonies evenly distributed over all experimental plates to control for spatial and plate-based bias. Because of the strong dependence on initial cell density, we found the growth distribution during light exposure of control colonies to be asymmetric (Fig. 1h). Colonies with initial cell densities of 60 CDE or less varied more in light resistance, resulting in too many erroneous false-positive calls of light sensitivity for control colonies in this initial cell density range. Control colonies with moderate (61–120 CDE) and in particular high (≥ 121 CDE) initial cell densities showed a much more uniform growth during illumination. We therefore discarded mutant colonies with initial cell densities < 60 CDE and evaluated visible light toxicity only for mutants with moderate (61–120 CDE; 2026 mutants) and high (≥ 121 CDE; 1591 mutants) initial cell densities. Light sensitivity in these two categories were scored positive at day 6 of illumination, if the mutants reached less than 400 CDE or 900 CDE, respectively (scoring criterium I). The estimated false-positive rates based on the control strain and criterium I were 0% for the high confidence and 4.7% for the moderate confidence calls (Additional file 2: Table S1). Finally, to avoid erroneously scoring generally slow-growing deletion strains as light sensitive, we normalized the growth of each mutant under light exposure to its corresponding growth in the dark, and then compared the dark-normalized light sensitivity of each deletion strain to that of the internal controls (*n* = 384) on each plate. We required light-sensitive mutants to have at least a 75% reduction in this double-normalized light tolerance index compared to the control strain (scoring criterium II; Fig. 1j). Using these two scoring criteria, we called 205 and 267 mutants as light-sensitive at high and medium confidence, respectively.

### Validating genes required for growth in visible light

We validated a selected set of positive calls in a three-tier procedure. First, 22 high confidence, 52 moderate confidence, and 16 negative calls were re-evaluated using our high-throughput method, but at higher replication and as dilution series covering a range of initial cell densities (corresponding to a dilution-factor of 3, Additional file 1: Fig. S2A, B and Fig. S3). We confirmed 86% of the high and 69% of the moderate confidence calls; the false-positive rate thus appeared somewhat higher than predicted from the control strain data. Again, the toxicity of visible light clearly depended on initial cell densities and this dependency varied across genotypes (Additional file 1: Fig. S2C). Next, we validated the same selected set of strains using our confirmation assay but testing homozygous diploid rather than haploid deletion strains. We reasoned that potential background mutations should mostly be heterozygotic in these diploids and that associated recessive effects on light sensitivity therefore should be masked. We confirmed 70% of the validated light-sensitive haploid mutants in the diploid mutant collection. Gene-environment effects on growth are often ploidy dependent, which may explain some of the negative calls [23].

**Fig. 2:**
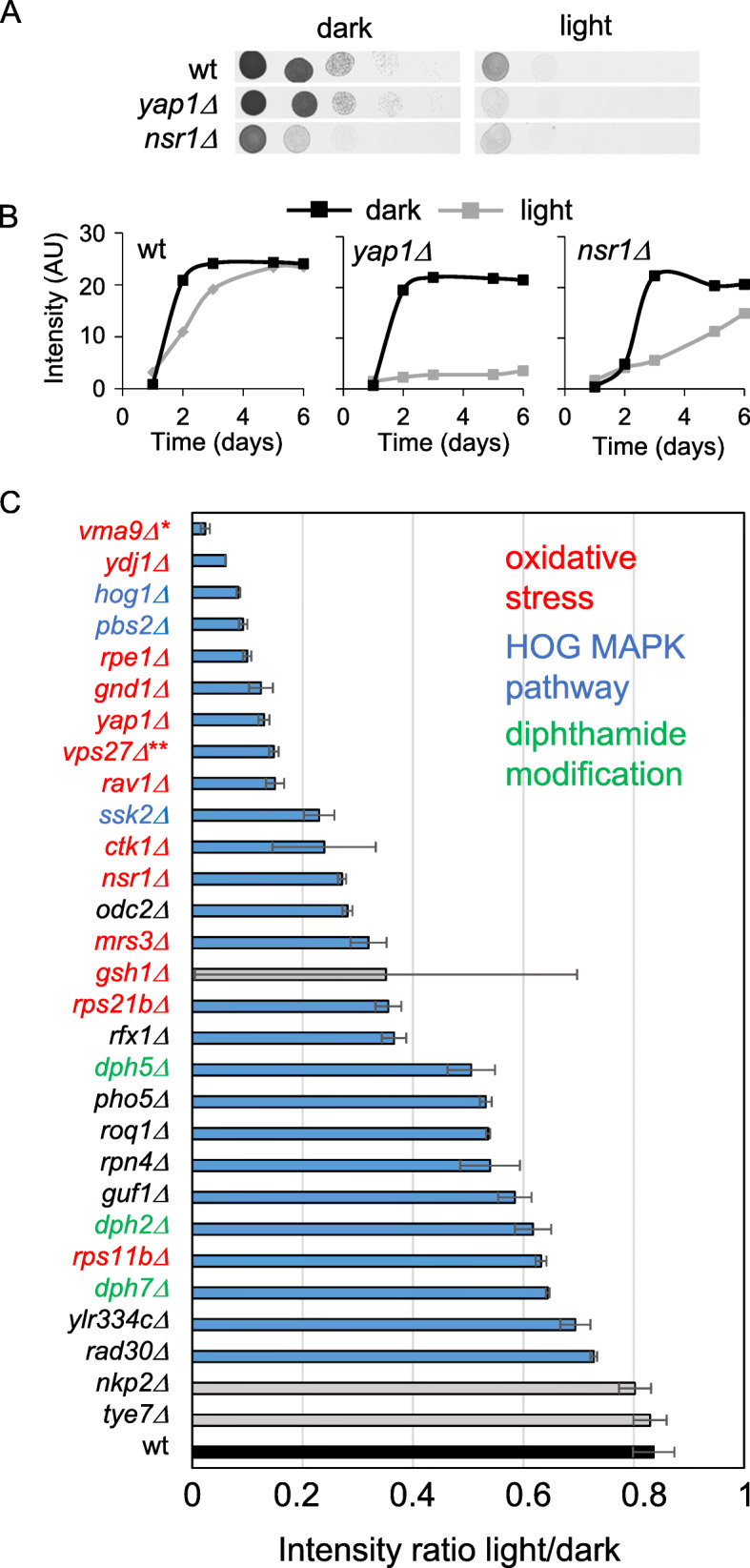
The second confirmation assay—quantitative serial dilution drop test. **a** Raw data drop test dilution validation assay. Strains were diluted to the same cell density (OD_610_~1) and then further diluted in 5 sequential 10-fold dilutions, and spotted on agar. Plates that were either exposed to light or kept in the dark were imaged daily. Images of the dark control and the light exposed plates after 2 days of incubation, exemplified by wild type, *yap1∆*, and *nsr1∆*. **b** Growth curves of the strains shown in **a** where the cell-spot intensity has been estimated for images taken over 6 days. **c** Ranked list of the most light-sensitive strains (Student *t* test; *p* < 0.05). Values represent averages from two independent experiments, and error bars indicate standard deviation (SD) (see Additional file 5 for individual values). Blue bars denote mutants displaying a statistically significant growth difference compared to the wild-type (*p* < 0.05) whereas gray bars indicate non-significant growth differences. The most light-sensitive mutants confirmed in the second confirmation assays show extensive overlap with mutants sensitive to oxidative stress (red names), the HOG MAPK pathway (blue names), and diphthamide modification of eEF2 (green names). The description of each of the gene deletions can be found in Table 1

Finally, we manually spotted pre-cultures of 34 of the homozygous diploid gene knockouts for genes showing a light defect in the primary genome-wide screen as standard dilution series (drop tests) onto solid media, making sure they had the same initial cell densities for optimal comparison of light sensitivity (Fig. 2a). We compared their growth under illumination to that of samples growing in complete darkness (Fig. 2b) and found 24 mutants (70%) to have significant (Student *t* test; *p* < 0.05) light-specific growth defects (Fig. 2c). We found that cells lacking the vacuolar H^+^-ATPase Vma9, the type I HSP40 co-chaperone Ydj1, and the osmoregulatory signaling components Hog1 and Pbs2, as well as the pentose-phosphate pathway enzymes Rpe1 and Gnd1, were more light-sensitive than the positive control Yap1 (Table 1).

**Table 1:** Functional descriptions of the validated most light-sensitive gene deletion (Student *t* test; *p* < 0.05) mutants in the second confirmation assay. The genes are listed according to the sensitivity ranking indicated in Fig. 2c. The GO slim terms indicated belong to the functional enrichment categories detailed in Fig. 3a, except for *VMA9*, *YDJ1*, *RPE1*, *GND1*, *VPS27*, *RAV1*, *ODC2*, *MRS3*, *PHO5*, *ROQ1*, *GUF1*, *YLR334C*, and *RAD30* which GO slim terms were not enriched. Descriptions are modified from SGD

Gene	GO slim	Description
*VMA9*	Ion transport (GO:0006811)	Data from mutant *ycl007c∆* that is a dubious ORF that overlaps with verified ORF *VMA9*, which is a vacuolar H+ ATPase subunit of the V-ATPase V0 subcomplex that is essential for vacuolar acidification
	Transmembrane transport (GO:0055085)	
*YDJ1*	Response to heat (GO:0009408)	Type I HSP40 co-chaperone involved in regulation of the HSP90 and HSP70 functions; involved in protein translocation across membranes; member of the DnaJ family
	Protein targeting (GO:0006605)	
*HOG1*	Response to osmotic stress (GO:0006970)	Mitogen-activated protein kinase involved in osmoregulation; mediates the recruitment and activation of RNA Pol II at Hot1p-dependent promoters
	Response to oxidative stress (GO:0006979)	
	Peptidyl-amino acid modification (GO:0018193)	
	Transcription by RNA polymerase II (GO:0006366)	
	Histone modification (GO:0016570)	
	Protein phosphorylation (GO:0006468)	
*PBS2*	Response to osmotic stress (GO:0006970)	MAP kinase kinase of the HOG signaling pathway involved in osmoregulation; activated under severe osmotic stress; mitophagy-specific regulator
	Peptidyl-amino acid modification (GO:0018193)	
	Protein phosphorylation (GO:0006468)	
*RPE1*	Generation of precursor metabolites and energy (GO:0006091)	D-ribulose-5-phosphate 3-epimerase, catalyzes a reaction in the non-oxidative part of the pentose-phosphate pathway.
*GND1*	Response to oxidative stress (GO:0006979)	6-Phosphogluconate dehydrogenase (decarboxylating), catalyzes an NADPH regenerating reaction in the pentose phosphate pathway
	Carbohydrate metabolic process (GO:0005975)	
*YAP1*	Response to oxidative stress (GO:0006979)	Basic leucine zipper (bZIP) transcription factor required for oxidative stress tolerance; activated by H_2_O_2_ through the multistep formation of disulfide bonds and transit from the cytoplasm to the nucleus
	Transcription by RNA polymerase II (GO:0006366)	
*VPS27*	Protein targeting (GO:0006605)	Data from mutant *ynr005c∆* that is a dubious ORF that overlaps verified ORF VPS27, which is an endosomal protein that is required for sorting of ubiquitinated proteins destined for degradation
	Endosomal transport (GO:0016197)	
	Ion transport (GO:0006811)	
*RAV1*	Cellular ion homeostasis (GO:0006873)	Subunit of the RAVE complex, which promotes assembly of the V-ATPase holoenzyme; required for transport between the early and late endosome/PVC
*SSK2*	Response to osmotic stress (GO:0006970)	MAP kinase kinase kinase of the HOG1 mitogen-activated signaling pathway involved in osmoregulation; also mediates actin cytoskeleton recovery from osmotic stress
	Protein phosphorylation (GO:0006468)	
*CTK1*	Peptidyl-amino acid modification (GO:0018193)	Catalytic (alpha) subunit of C-terminal domain kinase I (CTDK-I); phosphorylates both RNA pol II subunit Rpo21p and ribosomal protein Rps2p (the latter to increase translational fidelity).
	Transcription by RNA polymerase II (GO:0006366)	
	Protein phosphorylation (GO:0006468)	
*NSR1*	Ribosomal small subunit biogenesis (GO:0042274)	Nucleolar protein that binds nuclear localization sequences, required for pre-rRNA processing and ribosome biogenesis
	rRNA processing (GO:0006364)	
*ODC2*	Cellular respiration (GO:0045333)	Mitochondrial inner membrane transporter; exports 2-oxoadipate and 2-oxoglutarate from the mitochondrial matrix to the cytosol for use in lysine and glutamate biosynthesis
	Transmembrane transport (GO:0055085)	
*MRS3*	mRNA processing (GO:0006397)	Iron transporter that mediates Fe^2+^ transport across the inner mitochondrial membrane; active under low-iron conditions
	Transmembrane transport (GO:0055085)	
	Ion transport (GO:0006811)	
*RPS21B*	Ribosomal small subunit biogenesis (GO:0042274)	Protein component of the small (40S) ribosomal subunit
	Cytoplasmic translation (GO:0002181)	
	rRNA processing (GO:0006364)	
*RFX1*	Transcription by RNA polymerase II (GO:0006366)	Major transcriptional repressor of DNA-damage-regulated genes that recruits repressors Tup1p and Cyc8p to their promoters; involved in DNA damage and replication checkpoint pathway
*DPH5*	Cytoplasmic translation (GO:0002181)	Methyltransferase required for synthesis of diphthamide, which is a modified histidine residue of translation elongation factor 2
	Peptidyl-amino acid modification (GO:0018193)	
*PHO5*	Response to starvation (GO:0042594)	Repressible acid phosphatase.
*ROQ1*	Response to chemical (GO:0042221)	Ub-ligase substrate-specificity factor part of the stress-induced homeostatically regulated protein degradation (SHRED) pathway.
	Proteolysis involved in cellular protein catabolic process (GO:0051603)	
*RPN4*	Transcription by RNA polymerase II (GO:0006366)	Transcription factor that stimulates expression of proteasome genes
*GUF1*	Regulation of translation (GO:0006417)	Mitochondrial matrix GTPase that associates with mitochondrial ribosomes; important for translation under temperature and nutrient stress; may have a role in translational fidelity
*DPH2*	Cytoplasmic translation (GO:0002181)	Protein required for synthesis of diphthamide, which is a modified histidine residue of translation elongation factor 2
	Peptidyl-amino acid modification (GO:0018193)	
*RPS11B*	Ribosomal small subunit biogenesis (GO:0042274)	Protein component of the small (40S) ribosomal subunit
	Cytoplasmic translation (GO:0002181)	
	rRNA processing (GO:0006364)	
*DPH7*	Cytoplasmic translation (GO:0002181)	Diphthamide synthetase (standard name RRT2); required for last step of diphthamide biosynthesis, deletion leads to accumulation of diphthine, involved in endosomal recycling
	Peptidyl-amino acid modification (GO:0018193)	
*YLR334C*	Currently unannotated	Dubious open reading frame
*RAD30*	Mitotic cell cycle (GO:0000278)	DNA polymerase; involved in translesion synthesis during post-replication repair
	Chromosome segregation (GO:0007059)	
	DNA repair (GO:0006281)	
	DNA replication (GO:0006260)	

### Cellular processes required for growth in visible light

**Fig. 3:**
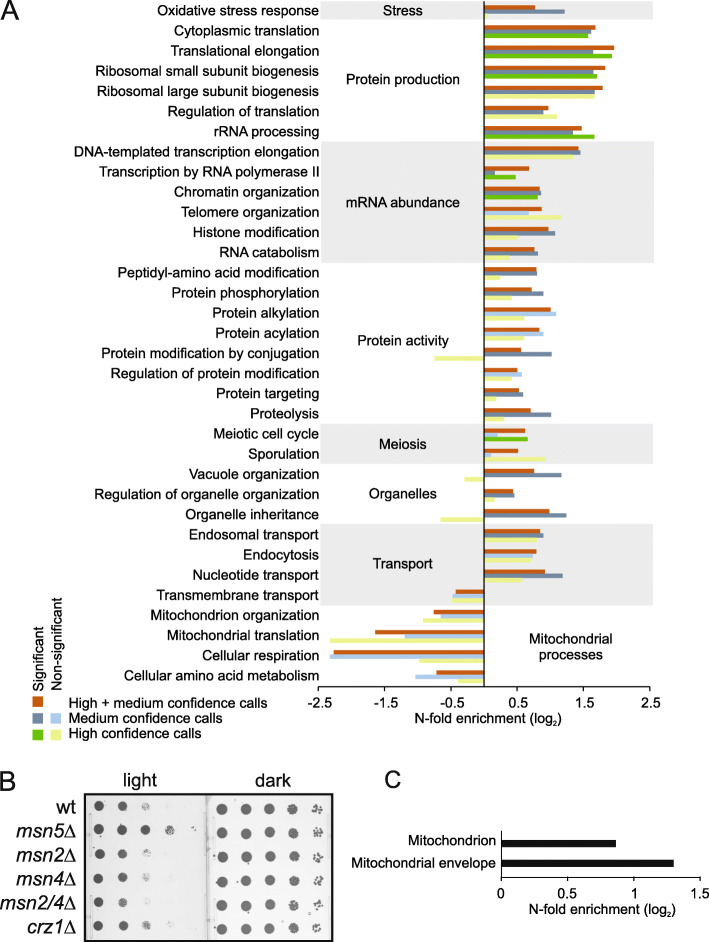
Functional characterization of light-sensitive mutants of all light-sensitive mutants in the initial screen. **a** Functional enrichment of GO slim categories (cellular process) among light-sensitive mutants called at high or medium confidence and among both sets combined (FDR, *q* = 0.06). Cellular processes with < 25 annotated genes were not evaluated. **b** Light sensitivity of strains deleted for *MSN2*, *MSN4*, *MSN5*, *CRZ1*, or both *MSN2* and *MSN4*. Pictures were taken after 3 days of exposure to light or of control cells incubated in the dark. A representative result from three independent experiments is shown. **c** Functional enrichment of genes annotated with GO slim categories indicating a mitochondrial function among strains showing improved growth at day 6 of illumination

We identified cellular processes enriched among light-sensitive mutants called at either high or medium confidence in our genome-wide screen (Fig. 3a). While the statistical power to call such enrichments differs somewhat between the sets, they tended to contain genes acting in the same cellular processes and therefore captured much the same underlying biology. This enhanced our trust in the medium confidence set. Thus, we found processes involved in transmitting information from DNA to active protein to be more common than expected by chance (FDR, *q* = 0.06; Fig. 3a). In particular, proteins linked to the ribosome and translation, directly in the form of ribosomal and translational components or indirectly in the form of genes participating in rRNA processing, were common. Because several of these, e.g., the ribosomal components Rps21b and Rps11b, were confirmed in the final validation assay (Fig. 2c), they reflect true links to photobiology rather than a general importance for growth typically associated with ribosomal mutations.

Genes involved in RNA polymerase II transcription, e.g., Tec1, a transcription factor that targets filamentation genes, and Gcr2, a transcriptional activator of glycolytic genes, were also required for growth in visible light. We have previously observed activation of the transcription factors Msn2, its functional homolog Msn4, and the Ca^2+^ activated Crz1 upon illumination [24, 25]. We therefore investigated the requirement for Crz1, Msn2, and Msn4 for growth in visible light (Fig. 3b). Only loss of both Msn2 and Msn4 impaired growth in visible light (Fig. 3b). In addition, deletion of the karyopherin Msn5 that causes the retention of Msn2 in the nucleus [26] resulted in enhanced light resistance, suggesting that nuclear Msn2/4 activity is important for cells to resist light stress.

Proteins of the oxidative stress response were also often required for growth in visible light (Fig. 3a), in line with that light exposure generates reactive oxygen species (ROS) [11, 12]. We therefore investigated which of the validated light-sensitive mutants that also have been linked to oxidative stress sensitivity [27–30]. We found 12 out of the 26 top hits (and 6 out of the top 10) to be required for growth under H_2_O_2_ or superoxide exposure (Fig. 2c). Two pentose phosphate pathway mutants, *gnd1∆* and *rpe1∆*, were among the most light-sensitive mutants (Fig. 2c; Table 1). The pentose pathway is a NADPH regenerating mechanism that is essential for growth under superoxide (menadione) stress [30].

The mutants *hog1∆*, *ssk2∆*, and *pbs2∆*, lacking key components in signaling via the high-osmolarity glycerol response (HOG) pathway, were all validated in our most stringent confirmation assay (Fig. 2c; Table 1). While osmotic stress is the major signaling stimulus for the HOG pathway, it is activated and important also under heat [31], citric acid [32], and cell wall stress [33, 34] as well as in response to particular forms of oxidative stress [35]. We add visible light to the list of stresses for which HOG components are required for growth.

Finally, respiration defective mutants have earlier been reported to be light-resistant [18]. We found mitochondrial and respiratory defective mutants to be underrepresented among light-sensitive mutants (Fig. 3a, “mitochondrion organization,” “mitochondrial translation,” and “cellular respiration”). Furthermore, mutants that grew clearly better than the control during light exposure, i.e., being light-resistant, were enriched for proteins located to the mitochondria and the mitochondrial membrane (FDR, *q* = 0.06; Fig. 3c).

### Diphthamide modification of His699 on eEF2 did not alter light absorbance

The elongation factor eEF2 is an essential enzyme in protein synthesis that contains a unique modification of a histidine (His699 in yeast; His715 in mammals) into diphthamide (DTA), obtained via 3-amino-3-carboxypropyl (ACP) and diphthine (DTI) intermediates [36]. The diphthamide modification is made in a four-step biosynthetic pathway that involves seven enzymes (Dph1-Dph7, [36, 37]). We found cells lacking three components required for the stepwise formation of the diphthamide modification to be sensitive to visible light, i.e., *dph2∆*, *dph5∆*, and *dph7/rrt2∆* (Fig. 2c). Although the functional role of the diphthamide modification is poorly understood, cells lacking *DPH2* or *DPH5* show an increase in − 1 frameshifting during translation. This suggests that they are required for translational fidelity [38], although this translational defect incurs no growth defects under optimal conditions [39]. We hypothesized that the His699 diphthamide intermediates, which are formed in the mutants when the modification is halted mid-process, absorb visible light and that their excited state might catalyze deleterious photochemical reactions, leading to the growth defects of the deletion mutants. To investigate this, we predicted the absorption spectra of the different modified His699 intermediates in eEF2-diphthamide biosynthesis with a combination of homology modeling, molecular dynamic (MD) simulations, *in silico* mutagenesis, and time-dependent density functional theory (TD-DFT) calculations. The atomic arrangements in eEF2 suggested that three closely positioned His-residues, His583, His694, and the modified His699, could form a “π-interacting triad” that might alter light absorption (Additional file 1: Fig. S4). However, from our *in silico* analysis this appeared not to be the case. We established the response to visible light in different DPH-mutants (*dph2∆*, *dph5∆*, *dph7∆*) by computing absorption spectra on a range of snapshot geometries from the trajectories of the modified intermediates of diphtamide-EF2. When His699 is unmodified, the three histidine residues remained at a distance that varied very little over time (absorbing light at wavelengths < 250 nm), indicating that His583, His694, and His699 do not really form a π-triad in such a way as to affect the absorption spectra in the visible range (Additional file 1: Fig. S5, S6). In addition, for the two intermediates DTI and DTA the lowest-lying absorption occurs at ~ 250 nm, similar to the system with unmodified His699, proving that the light-absorption of the His699 variants is most likely not involved in the DPH-mutants’ light sensitivity. The ACP intermediate can absorb low energy UV light (340 nm), but not in the visible range (Additional file 1: Fig. S6). Thus, we conclude that it is not the absorption of visible light of these intermediates that is the cause of the DPH-mutants’ light sensitivity. One possibility for their light sensitivity could be a role of fully diphthamide-modified eEF2 in proper translation of some specific proteins required for light resistance. Alternatively, the enzymes involved in the diphthamide pathway might have alternative functional roles, besides modifying His699 of eEF2.

### The sensing of visible light

**Fig. 4:**
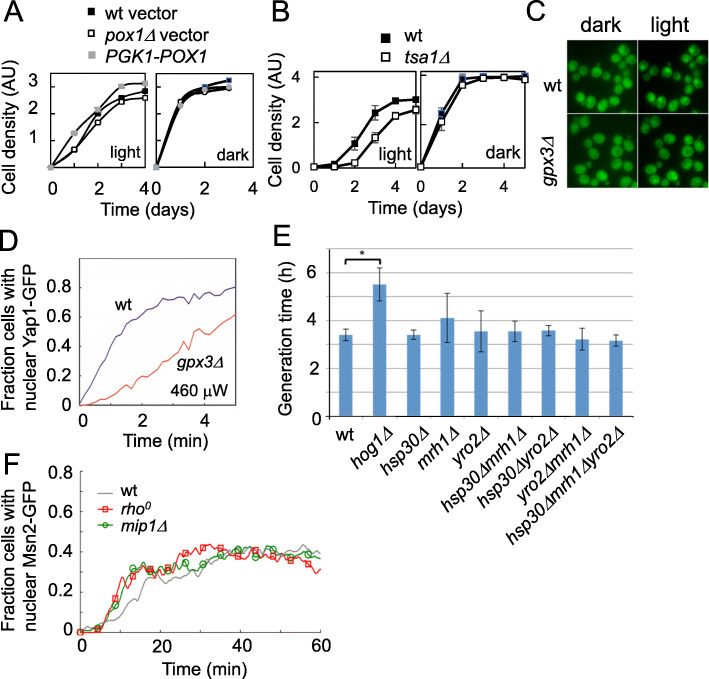
Light sensitivity in relation to different potential light-sensing mechanisms. **a** Light sensitivity of cells lacking Pox1 (*pox1Δ*) or overexpressing *POX1* (PGK-*POX1*) as assayed by the spot-test assay and growth curves. A representative result from two independent experiments is shown (see Additional file 5 for individual values). **b** Light sensitivity of cells lacking Tsa1 (*tsa1Δ*) as assayed by the spot-test assay and growth curves. Values are averages from two independent experiments, and error bars represent SD. **c** Yap1-GFP localizes to the nucleus in response to light exposure. Left: cells exposed to 460 μW constant light exposure for 1 min wt or *gpx3Δ*. Right: cells before exposure to light. **d** Quantification of Yap1-GFP nuclear localization. The data presented represent averages of localization scored in individual cells, *n* = 246 (wt) and *n* = 152 (*gpx3∆*). **e** Generation times for the indicated opsin-like single deletion (*hsp30Δ*, *mrh1Δ*, *yro2Δ*), double deletion as well as a triple mutant strain. As a positive control the light sensitive *hog1Δ* strain was used. Values represent averages of at least three independent experiments, and error bars indicate SD (see Additional file 5 for individual values). Only the *hog1∆* strain showed a significant (Student *t* test, p < 0.05) difference to the wt strain (indicated with *). **f** The Msn2-GFP nuclear localization during light exposure in two respiratory deficient mutants; the *rho*- and the *mip1∆* strain. Values are averages from analysis of individual cells; *n* = 112 (wt), *n* = 98 (*mip1∆*), and *n* = 58 (rho^0^). *MIP1* encodes an essential component of the RNA polymerase for transcription of genes in the mitochondrial genome. Msn2-GFP nuclear localization trajectories for a *rho*^*0*^ and a *mip1Δ* strain indicate that respiratory activity, and the corresponding H_2_O_2_ generation in the mitochondria, is not required for the Msn2 nuclear localization response to light

Next, we explored the role of light sensing functions in the resistance to visible light. We have previously noted a key importance of the peroxisomal acyl-CoA oxidase Pox1 in light sensing in response to blue light, where the flavin-linked Pox1 converts light into a H_2_O_2_ signal that is sensed by the peroxiredoxin Tsa1 which, in turn, transmits this information to the stress-dependent transcription factor Msn2 and stimulates its nuclear accumulation [14]. We therefore examined the growth of *pox1Δ* and *POX1* overexpressing cells (PGK1-*POX1*) during illumination (Fig. 4a); *pox1Δ* was somewhat light sensitive and PGK1-*POX1* somewhat light resistant. This is in line with a moderate role of Pox1 in visible light resistance. We next investigated whether the peroxiredoxin Tsa1, which functions both as a H_2_O_2_ receptor [14] and a thiol-peroxidase [40, 41], was important for light resistance (Fig. 4b). Cells lacking Tsa1 grew slowly, and slower than *pox1∆*, during light exposure, supporting that ROS of non-Pox1 origin limit light growth.

The Yap1 transcription factor responds both to H_2_O_2_ as well as to secondary oxidation products of oxidative stress, such as the lipid peroxide 4-HNE [42, 43]. Supporting that Yap1 activity is required for cells to grow during illumination, we noted that a Yap1-GFP fusion protein rapidly accumulated in the nucleus upon illumination (Fig. 4c, d). Yap1 nuclear localization in response to transient H_2_O_2_ stress depends on the H_2_O_2_ receptor Gpx3 [43], whereas visible light activation of Yap1 was only partially Gpx3-dependent, reminiscent of the Yap1 response to lipid peroxidation (4-HNE), thiol-reactive compounds, and chronic H_2_O_2_ stress [42, 43].

We also explored if the three opsin-like yeast proteins, Hsp30, Mrh1, and Yro2, contributed to light resistance, although these proteins lack a lysine residue believed to be necessary for retinal binding [13]. Single, double, and triple mutants of these opsin-like genes all had normal light growth (Fig. 4e) and therefore no evident role in light resistance.

Because respiration-defective mutants are light resistant, we next investigated potential light sensing via respiration impinging on the Msn2-GFP nucleocytoplasmic shuttling during light exposure [14, 24, 44]. However, neither non-respiratory *rho*^*0*^ nor *mip1∆* (*MIP1* encodes the catalytic subunit of the yeast mitochondrial DNA polymerase) strains, both missing mitochondrial DNA and thus devoid of respiratory activity and mitochondrial ROS generation, showed altered Msn2-GFP nuclear localization during light exposure (Fig. 4f). Thus, neither cytochromes, active respiration, nor mitochondrial ROS production are required for Msn2-related light-signaling. Moreover, the light resistance of mitochondrial mutants is not a reflection of increased Msn2 activation/nuclear localization.

### Protein kinase A controls the yeast resistance to visible light

**Fig. 5:**
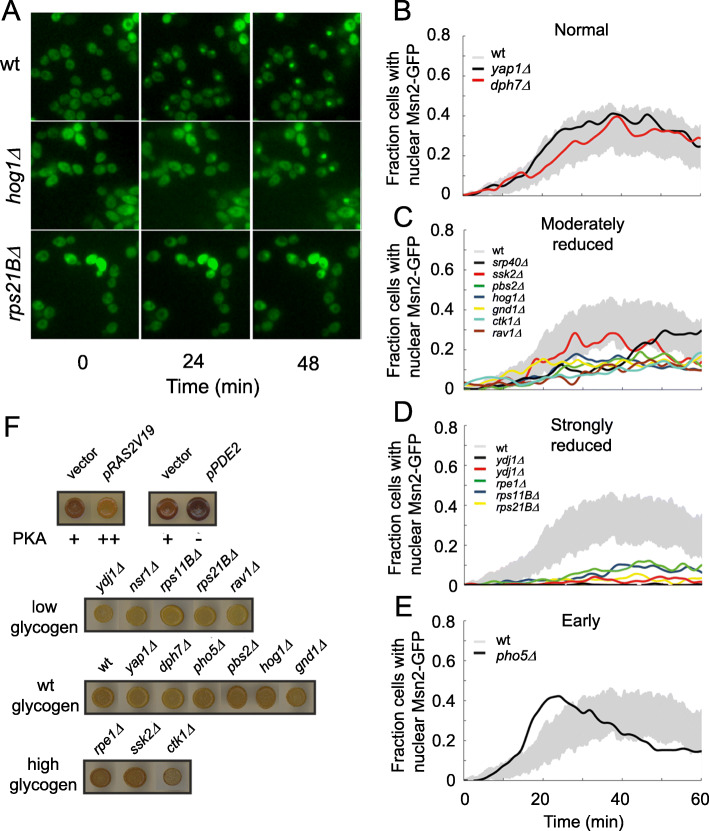
Indirect reporters of the level of PKA activity in the light-sensitive mutants. **a** Time-lapse micro-graphs of Msn2-GFP in wild type, *hog1∆*, and *rps21B∆*. **b**–**e** The total nuclear localization trace of each strain compared to the wild type. The gray area corresponds to the mean value of 15 individual experiments of the wild type strain plus/minus one standard deviation. Each wild type experiment contains 50–60 cells. The trace for each deletion strain is the combined response for > 99 cells (up to 170 cells) from 2 or 3 experiments on individual days. Data from the deletion strains have been smoothed using a spline smoothing algorithm in order to visualize the main trend for each individual strain. Msn2 nuclear localization for selected single deletion strains during continuous light exposure response has been divided into 4 groups. Many light-sensitive mutants display reduced Msn2-GFP nuclear localization in response to light. **b** Normal/wild type response. **c** Moderately reduced response where Msn2 spends less time in the nucleus. **d** Strongly reduced response, where Msn2p does not enter the nucleus and **e** early, but otherwise normal response, unique for *pho5Δ*. **f** Glycogen staining of cells from the indicated strains using iodine vapor. A representative result from at least two independent experiments is shown. In the top row, the wild-type transformed with a centromeric plasmid expressing hyperactive RAS2G19V (p*RAS2V19*) yielding constitutively high PKA activity (PKA ++), multicopy *PDE2* (p*PDE2*; phosophodiesterase) resulting in low PKA activity (PKA −), or the corresponding vector controls (vector, intermediate PKA activity +) are shown. The three remaining rows display glycogen staining of the indicated light-sensitive deletion mutants classified as staining similar to the wild-type (wt glycogen), lower than the wild-type (low glycogen), or higher than the wild-type (high glycogen)

The wide variety of functional categories represented by the light-sensitive deletion mutants (Fig. 3a, Table 1) urged us to search for common denominator(s). Msn2 nuclear localization in response to visible light is amplified by low, and attenuated by high, activity of protein kinase A [14, 44]. Light-induced Msn2 nuclear localization therefore reports on PKA activity. We transformed Msn2-GFP into 14 light-sensitive deletion mutants (Fig. 2c) to probe Msn2 nuclear localization and PKA activity in these strains. We exposed cells to blue light (450–490 nm) and the Msn2-GFP nucleocytoplasmic shuttling was followed using time-lapse fluorescence microscopy (Fig. 5a). At the chosen low light intensity, Msn2-GFP oscillates in most mutant cells with no extended nuclear localization [14, 44]. We distinguished four mutant categories: normal (Fig. 5b), moderately reduced (Fig. 5c), strongly reduced (Fig. 5d), and early but otherwise normal (Fig. 5e) Msn2 nuclear localization. Because most mutants showed moderately or heavily reduced Msn2 nuclear localization, we tentatively concluded that PKA activity also is elevated in these cells under light exposure.

We next analyzed glycogen accumulation, an often used proxy for PKA activity [45, 46]. Yeast accumulates glycogen in response to low PKA activity, whereas high PKA activity, e.g., in the mutant *RAS2V19* (Fig. 5f) reduces yeast glycogen accumulation. We found *ydj1∆*, *nrs1∆*, *rps11b∆*, *rps21b∆*, and *rav1∆* to have low levels of intracellular glycogen, in line with these strains having high PKA activity (Fig. 5f), thus supporting our data on Msn2-GFP nuclear localization.

**Fig. 6:**
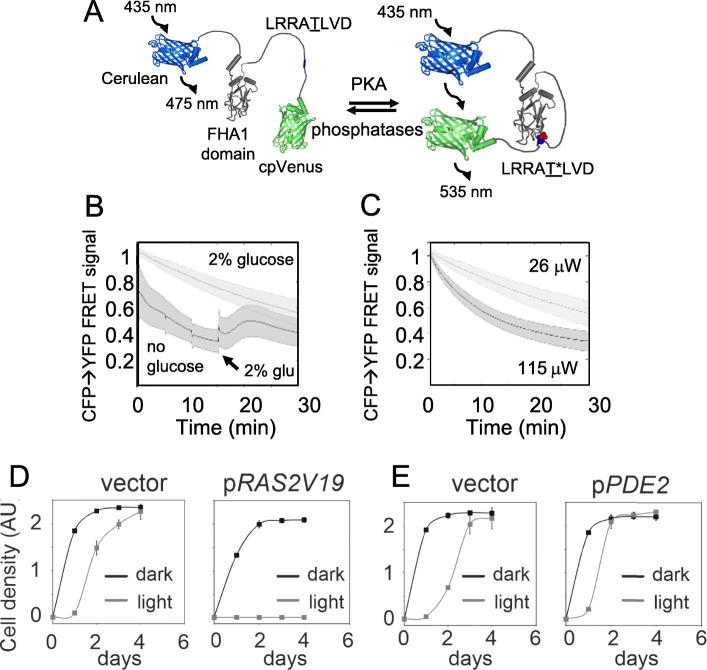
Reduced protein kinase A activity is required for the resistance to visible light. **a** Design of the AKAR4 PKA FRET sensor. Adopted from [48]. **b** PKA activity in response to glucose starvation and glucose re-addition, as measured using the AKAR4 PKA sensor in individual cells; *n* = 44 (control) and *n* = 68 (glucose starvation + re-addition of glucose). Error bars = SD. **c** PKA activity in response to different levels of light intensity, as measured using the AKAR4 PKA sensor in individual cells; *n* = 44 (26 μW) and *n* = 117 (115 μW). Error bars indicate SD. **d**, **e** Growth curves of strains with altered protein kinase A activity in absence (black lines) or presence (gray lines) of light. Averages from two independent experiments are displayed. Error bars indicate SD (see Additional file 5 for individual values)

To investigate directly to what extent illumination affected PKA activity, we used the ectopic FRET-based PKA sensor AKAR4 [47]. In this reporter, an Fha1 domain containing a PKA site (LRAT*LVD) is placed in between CFP and YFP and upon its phosphorylation FRET between CFP and YFP occurs (Fig. 6a). We verified that the sensor measures PKA activity in yeast by starving cells for glucose; this resulted in an instant, dramatic reduction in the FRET signal that was restored upon glucose re-addition (Fig. 6b). Using this reporter, we observed a light intensity-dependent, gradual reduction in PKA activity (Fig. 6c). This agrees with the gradual accumulation of H_2_O_2_ that we previously reported to be required for PKA-dependent light signaling [14]. Finally, we tested directly to what extent altered PKA activity affected the ability of cells to grow in light. We examined mutants with constitutively high (p*RAS2V19*; a constitutively active RAS mutant) or low (p*PDE2*; overexpression of the phosphodiesterase Pde2 reducing cAMP levels) PKA activity. We found a strong correlation between PKA activity and poor growth in visible light, with the high PKA (*pRAS2V19*) strain failing to grow upon illumination (Fig. 6d) and the low PKA strain (p*PDE2*) growing better than the wild type (Fig. 6e). We therefore conclude that a low PKA activity is required for growth in visible light.

## Discussion

### Yeast photobiology is multifaceted and complex

We extended the list of genes required for normal growth under visible light stress to include a rich and diverse flora of functional features. This points towards more complex and diverse photobiology in non-photosynthetic organisms than previously envisaged. To our knowledge, this is the first genome-wide screen to systematically identify important resistance-mechanisms during growth in visible light in any organism.

Previous studies on light resistance suggested that flavins and porphyrins are the main initial molecular targets for visible light in yeast [49]. The activities of the multi-subunit porphyrin-containing respiratory components cytochrome bc1 and cytochrome oxidase appear to be sensitive to light over a wide range of the visible spectrum, with cytochrome oxidase being most sensitive and most responsive to irradiation in the 400–450 nm range [17]. Moreover, respiratory-deficient petite yeasts (*rho*) lacking cytochromes are resistant to light intensities that cause photokilling of wild-type cells [18]. We also confirmed mitochondrial and respiratory defective mutants to be underrepresented among light-sensitive and enriched among light-resistant mutants (Fig. 3). Flavoproteins are ubiquitous proteins involved in a wide variety of biological redox processes ranging from redox-based catalysis to DNA repair [15]. The absorption of visible light by flavoproteins is also affected by their oxidation state; the yellow oxidized state of the flavoprotein glutathione reductase exhibits absorption maxima around 360 and 455 nm, while upon reduction the enzyme turns red and the absorbance maxima rises to around 530 nm [49]. One of the fundamental properties of flavin is its ability to readily react with molecular oxygen, and flavin-dependent oxidases use dioxygen as an electron acceptor to generate H_2_O_2_. We have previously shown that a flavin-binding protein, the peroxisomal oxidase Pox1, plays a key role in yeast blue light sensing through the light-induced production of H_2_O_2_ [14]. However, cells lacking or overexpressing *POX1* differed only moderately from wild-type cells in their ability to grow under light stress (Fig. 4a). This suggests that other proteins and/or chromophores participate in light sensing and trigger a cellular response that is adequate for near normal growth under light stress. We investigated the possible involvement of the opsin-like yeast proteins Hsp30, Mrh1, and Yro2, in light resistance but found no effect on the ability to grow upon illumination of their ablation in either single, double, or triple mutants (Fig. 4e). In addition, cells lacking mitochondrial DNA and thus cytochromes as well as active respiration did not affect light-dependent Msn2 signaling (Fig. 4f).

Light can alter the conformation of flavin-proteins, leading to their mis-folding, aggregation, and ultimately degradation [16, 50, 51]. We found support for a light-driven protein aggregation in the strong light sensitivity of a strain missing the HSP40 co-chaperone Ydj1, which regulates the chaperones Hsp90 and Hsp70. We also found Roq1, which is involved in protein quality control, and Vps27, which sorts ubiquitinated proteins destined for degradation in the vacuole, to be among the proteins most important for growth under light stress (Fig. 2c). Taken together, the importance of these genes points to light-induced protein aggregation as an interesting avenue for future investigations.

### Protein kinase A controls the response to visible light

We showed that illumination lowers the activity of protein kinase A and that this reduced PKA activity is key for cells to grow well in the presence of visible light. We also found that many light-sensitive mutants exhibited abnormally high PKA activity. This suggests that an inability to keep PKA activity sufficiently low for an adequate response to light stress may explain their growth defects. Light is the most important stimulus for the circadian clock, which empowers organisms, from cyanobacteria to humans, to maintain autonomous cycles corresponding to day and night even in complete darkness. Protein kinase A signaling has previously been identified as a core circadian clock mechanism in mammals [52]. Thus, the role of protein kinase A in photobiology is conserved from yeast to mammals. Our results raise the possibility that it also in multicellular organisms functions in the defense against light stress and not only in circadian clock regulation. The key regulatory role of PKA under light stress in yeast matches its central importance in the resistance to oxidative stress [53, 54]. A close connection of ROS, responses to light, and the circadian clock has been found in zebrafish, where H_2_O_2_ was suggested to act as a second messenger by which light controls circadian rhythms [55]. It is thus quite possible that protein kinase A serves to mediate this signal. In relation to this it is interesting to note that we recently reported H_2_O_2_ to inhibit the activity of protein kinase A through Tsa1-dependent direct redox-regulation of a conserved cysteine in the substrate-coordinating activation loop in the catalytic subunit Tpk1 [54]. Thus, the increased H_2_O_2_ documented upon illumination of cells [14] is expected to similarly repress PKA activity without exerting a major effect on cAMP levels.

Protein kinase A is a key regulator of many other processes, including memory and hormone responses in mammals [56]. Moreover, it controls cellular decisions on proliferation versus defense and morphogenesis in baker’s yeast, as well as in other fungal species [57–59]. In relation to light, previous studies in the fungus *T*. *atroviride* suggest key roles of both regulated ROS-production (through NADPH-oxidase proteins) and of cAMP and protein kinase A in photo-conidiation in response to blue light [60, 61]. Thus, the close connection between PKA and oxidants in regulating light responses might well be conserved.

### The role of ROS production in the response to light

A key role for ROS in the response to light is supported by the increased light resistance of cells experiencing anaerobiosis, i.e., metabolism in absence of oxygen and therefore in the near absence of ROS [62]. We also noted a high overlap in our verified list of light-sensitive mutants (Fig. 2), with yeast mutants previously found to be sensitive to oxidative stress [27–30]. In this context, it is interesting to note that expression analyses of the light responses of the fungi *Aspergillus nidulans* and *Neurospora crassa*, which express dedicated red- (phytochromes) and blue-light (White Collar complex) sensing mechanisms, respectively, revealed many oxidative stress-related genes to be activated by light [63, 64]. Similarly, transcriptome analyses in the soil plant symbiont fungus *Trichoderma atroviride* showed that a significant proportion of the light-induced genes are related to oxidative and other types of stress responses [65], suggesting an overlap between yeast photobiology and light responses in fungi with dedicated light receptors.

Two recent studies came to different conclusions regarding the importance of ROS in the yeast light response [14, 19]. On the one hand, Robertson et al. found that the sensitivity of the yeast metabolic cycles to light was similar in wild-type cells and in cells lacking the Yap1 transcription factor required for the expression of several anti-oxidants. The authors concluded that light-induced ROS production alone could neither account for the altered period of the yeast metabolic cycle nor its amplitude during exposure to light. However, the authors did not try to specifically modulate the cycles by directly adding ROS. Given the redundancy of the yeast anti-oxidant responses (see e.g. [66]) and the fluctuations in ROS expected from altered oxygen consumption [67], the question on what effect ROS may have on the yeast metabolic cycles still remains open. In contrast, we recently showed that the response of yeast PKA to light, controlling nuclear localization of Msn2, could be recapitulated entirely through the addition of exogenous H_2_O_2_ [14], thus supporting a key role of ROS in the response of yeast to visible light.

### Light resistance is not exclusively dependent on PKA

Absence of the sister transcription factors Msn2 and Msn4, major signaling outputs downstream of PKA, induced only a moderate growth defect in light. Thus, although Msn2 might be a good reporter for studies concerning light sensing and the initial light response [14, 24, 25, 44, 68, 69], other pathways clearly have important roles in transmitting visible light signals. The HOG MAP kinase pathways is one of these, since mutations in the vital signaling pathway components *HOG1*, *PBS2*, and *SSK2* resulted in strong light sensitivity (Fig. 2). This sensitivity could conceivably stem from PKA being induced to compensate for the absence of HOG signaling. However, because these mutants accumulated glycogen at levels similar to that of wild-type cells (Fig. 5f) and because Msn2 nuclear localization decreased only moderately in HOG mutant strains upon exposure to light (Fig. 5c), both being consistent with at the most limited PKA activation, this can only be part of the explanation. Interestingly, using global transcriptomic analysis and chemical inhibition of alleles of the Pbs2 and Tpk1/2/3 kinases, antagonistic regulation of HOG and PKA-dependent targets under a wide variety of different stress conditions was recently reported [70]. The observation that Tpk1/2/3 inhibition appeared to be epistatic to Pbs2-deficiency for inducing the Msn2/4 target *HSP12* was taken as evidence for HOG signaling negatively regulating PKA. This is consistent with a direct link between HOG and PKA in light sensitivity. Notable in relation to MAPK signaling, the key regulator of the responses of *N*. *crassa* to blue light and circadian rhythms, the White Collar complex, has been shown to cyclically regulate the activity of the stress-activated MAPK OS-2 in response to clock input through binding to the promoter of the MAPKKK OS-4 [4]. In addition, red light sensing through the phytochrome FphA has been noted to activate the stress-activated MAPK SakA in the fungus *A*. *nidulans* and the entire SakA MAPK cascade was picked up in a screen for genes required for expression from a light-regulated promoter [71]. This supports a key role of a MAPK cascade in responses to red light in this fungus.

Another route that seems to act independently of PKA is the importance during light growth of the biosynthetic systems for diphthamide modification of the specific histidine residue 699 on the translational elongation factor eEF2. The functional importance of this modification is not fully understood [39] but it appears to play a role in translational fidelity [38]. We showed a strong link to light resistance of this highly selective post-translational diphthamide modification, which will open new avenues for functional investigations. Interestingly, it has been reported that loss of diphthamide modification (*dphΔ*) on eEF2 combined with eEF2 undersupply (*eft2Δ*) causes a synthetic growth phenotype in the composite mutant (*dphΔeft2Δ*) [72, 73]. Thus, the diphthamide modification becomes crucial for yeast cells upon eEF2 downregulation. The role for eEF2 in stress tolerance is underscored by the fact that eEF2 mutants are sensitive to oxidative stress [74]. We hypothesize that light-induced production of ROS impairs eEF2 function and that the corresponding reduced translational capacity is particularly problematic if translation also is error-prone, as in *dphΔ*. In the context of fully functional translation, it should also be stressed that the activity of five of the proteins for tRNA modification (tRNA dihydrouridine synthase, *DUS1-4*) are dependent on binding of the flavin FMN [15], thus potentially being light targets in the visible range. Light-triggered destruction of these tRNA dihydrouridine synthase enzymes could hamper tRNA uridylation and be an additional factor negatively affecting translation.

### Light as a selection pressure during yeast evolution

The large number of light-sensitive mutants raises the question as to what extent light has been a natural selection pressure for yeast and other non-photosynthetic organisms. We conducted our growth screen at a light intensity of 35 W/m^2^. To set this intensity in perspective, a cloudy day would correspond to about 100 W/m^2^, while a sunny day can go up to 1000 W/m^2^. Thus, our screen was performed at light intensities that organisms in nature very frequently are exposed to. This makes it likely that natural light exposure has been a common, strong selection pressure for many organisms living in light-exposed niches. Bohnerts and co-workers found yeasts to have developed increased peroxide tolerance on the sunny versus the shady side of the Evolution Canyon in Israel [75], providing an ecological connection between light exposure and oxidative stress. The rich variety of light-dependent protective systems suggests that light adaptation has played an underestimated role in shaping the biology of non-photosynthetic organisms.

## Conclusion

Our genome-wide analysis shows that a wide array of functions is important to handle the negative effects from visible light. This points towards a more complex and diverse photobiology in non-photosynthetic organisms than previously envisaged. Flavin- and porphyrin-binding proteins are believed to be the main targets to absorb visible light leading to malfunction of some of these proteins. Indeed, we have earlier shown that a flavin-binding protein, the peroxisomal oxidase Pox1, plays a key role in yeast blue light sensing through the light-induced production of H_2_O_2_ [14]. However, we report here that cells lacking or overexpressing *POX1* differed only moderately from wild-type cells in their ability to grow in visible light. Thus, we propose that other proteins participate in light sensing and growth under light stress, and we report yeast photobiology to be multifaceted and to involve genes functioning in signaling, transcription, protein translation, protein aggregation, and oxidative stress defense.

Moreover, we find that a common denominator that controls many of the various responses to visible light is the central signaling component protein kinase A. We show that illumination lowers the activity of protein kinase A. In addition, this reduced PKA activity appears vital for cells to grow in visible light since many light-sensitive mutants exhibited abnormally high PKA activity. Thus, we add visible light to conditions for which there is an important regulatory role of PKA and raise the possibility that this conserved regulatory component also functions in the defense against light stress in multicellular organisms.

We hope that our study will open up new avenues for analyses of the effects of visible light and lead to a better awareness of its effect on even non-photosynthetic organisms and their evolution. In addition, yeast researchers might need to reconsider how to grow and study yeast in the laboratory, since even rather moderate intensities of visible light might influence the results.

## Methods

### Yeast strains used in screens and confirmation assays

The *S*. *cerevisiae* haploid strain BY4741 (*MAT*a *his3*∆*1 leu2*∆*0 met15*∆*0 ura3*∆*0*) and diploid strain BY4743 (*MAT*a/*MAT*α *his3*∆*1*/*his3*∆*1 leu2*∆*0*/*leu2*∆*0 met15*∆*0*/*MET15 LYS2*/*lys2*∆*0 ura3*∆*0*/*ura3*∆*0*]), and their corresponding genome-wide gene-deletion collections (*ORF::kanMX4*) of 4700 strains for non-essential genes, were used in the phenotypic screens. In the initial genome-wide screen, the haploid deletion BY4741 collection was examined. Positives from the first screen were then re-tested, both as haploid (BY4741) and diploid (BY4743) gene knockouts, in a subsequent validation screen. A subset of genes confirmed as required for growth in visible light was finally probed again using serial dilution drop tests of the diploid (BY4743) gene deletions.

Transcription factor- and *pox1Δ*-single mutants were from the BY4742 (*MAT*alpha *his3*∆*1 leu2*∆*0 lys2*∆*0 ura3*∆*0*) haploid deletion collection whereas strains YMM114 [[53], genotype BY4742 *tsa1Δ::natMX4*] and YMM174 [[54], genotype BY4742 *msn2Δ::hphMX4 msn4Δ::natMX4*] were also used. Overexpression of *POX1* was achieved from the strong, constitutive *PGK1* promoter [plasmid pRS316-PGK-*POX1* [76]] in strain BY4742, whereas the vector controls were transformed with plasmid pRS316 [77]. BY4742 was used for the construction and control for the analyses of the opsin-like genes, and all the constructed mutants were confirmed to have the same genotype as BY4742 by plating on selective media.

### The primary genome-wide screen for light-sensitive mutants

We first tested both agar-to-agar and liquid-to-agar transfer and found the latter to give the best signal-to-noise ratio when scoring light-sensitive mutants (data not shown). We performed the liquid-to-agar pinning in the following way: after stationary incubation in flat-bottom 96-wells microtitre plates for 48 h at 30 °C in 300 μl liquid YPD medium (2% glucose, 20 g/L peptone, 10 g/L yeast extract), stationary phase cultures were pinned onto agar plates. Prior to pinning, plates were shaken at 500 rpm (Vibrax VXR basic, IKA, Germany) for 10 min to ensure proper mixing and optimized cell transfer. Strains were pinned from liquid medium using 96-format long-pin pads onto solid agar plates in a 384-array using a pinning robot (RoToR HDA; Singer Instruments, UK). Agar plates contained synthetic complete medium (0.17% yeast nitrogen base, 0.5% ammonium sulfate, 2% [wt/vol] glucose, and 0.062% complete supplement mixture with all amino acids, plus 1% succinic acid and 0.6% NaOH to buffer the medium to pH 5.8).

All pinning, both for agar-to-agar and liquid-to-agar transfer, was conducted using the default version 1 of the pinning program, except for the first confirmation assay (see below). The pinning pressure was 7% with an agar overshoot of 2 mm and a pinning speed of 19 mm/s, and no agar mixing was applied. Each colony was pinned once with a pad pickup pressure of 80%. For the primary genome-wide screen, the type of pinning was liquid-to-agar pinning from four 96-format plates with strains in liquid medium to one 384-format agar plate. In each of the experimental plates, 76 controls (*his3∆*) were included that later on would be used for growth scoring (setting scoring thresholds and to estimate the number of false-positive; see below).

The experimental set-up for the primary screen is shown in Fig. 1a, b. The stand and the thin wooden ribs holding the plates (Fig. 1b) facilitated good air circulation around the plates to maximize heat conductance and cooling, circumventing the potential problem of heating from the light source and in this way keeping the temperature of the plates at ~ 30 °C. To prevent dehydration from the agar, each lid-edge was closed with parafilm. To increase the intensity and to get an even distribution of the light irradiation, a white paper was fitted to the outside bottom of each plate. In order to minimize UV light induced DNA damage, the light source consisted of three UV radiation-filtered fluorescent lamps (Osram L940, 58 W) in a standard lamp fixture equipped with a reflector. Figure 1a shows the spectrum of the Osram L940 fluorescent lamp in comparison to a commonly used non-UV filtered fluorescent lamp in the lab. The spectra were measured directly from the lamps using a fiber-coupled spectrometer (QE65000-FL, Oceans Optics). Spectra from either of the lamps were collected with and without the lid in front of the fiber. The ratio of the two spectra from each lamp yielded a flat line at 0.9 over the whole spectral range, meaning that the plastic in the lid did not modify the overall spectrum of the lamp, but reduced light intensity evenly with 10%. The plates were placed 20 cm from the light source with the top (lid-side) up on ribs on a stand and the cells were irradiated through the lid of the plates with an on-site intensity of ~ 35 W/m^2^. The distance between the lamp and the plates was optimized in such a way that we got a substantial contrast in growth between the wild type and the positive control *yap1∆*, the only deletion strain previously reported to be sensitive to visible light (Fig. 1c, d, [19]). The control plates (no illumination) were wrapped up in aluminum foil and kept in the dark at 30 °C. After 0, 1, 2, and 6 days, the plates were imaged using a 12.3-megapixel digital camera (D5000, Nikon, Japan). For example images from the genome-wide screen for the time points used in the scoring of light sensitivity in dark and light, see Additional file 1: Fig. S1.

### The primary genome-wide screen—image analysis and data extraction

The light sensitivity of the mutants in the genome-wide screen was evaluated using a set of image analysis algorithms implemented in MATLAB®. The overall design of the image analysis method is based on calculating the cell density. Thus, it should be emphasized that our scoring algorithm is based on colony intensity and not on colony area. The main reasons for this are twofold: to give a good estimate on the cell density for day 0 where after pinning all cell patches are of about equal size but might contain different amount of cells, and to circumvent the drastic spatial effects encountered during growth on plates where colonies on the edges reach much larger area [78].

Based on the pixel values in the image a cell density estimate (CDE) for each of the 384 colonies per plate was calculated, corresponding to the sum of the pixel-intensity of the colony subtracting the local background. Each row of 24 colonies (each row is 30 pixels high and 1440 pixels wide) were initially analyzed individually. An intensity profile over each row was computed by summing the top 25 pixel values for each column (the 30 pixels high column). The rational for extracting the sum of each column is to enhance the signal-to-background relation for faint colonies at the start of the experiment (day 0; Additional file 1: Fig. S1). The intensity profile over each colony was then extracted by stepping over the profile of summed values with a step of 60 pixels (corresponding to the center-to-center distance between the colonies) with the colony pixel values centered in each of the colony profiles (Fig. 1g). For each colony profile, the colony intensity was then calculated by taking the median of the top 10 pixel values of the central 20 pixel values. The background intensity was calculated by taking the mean of the minimum summed pixel values on the left and the right side of the colony (corresponding to pixel 5–20 and pixel 40–55) in the profile. The CDE was then calculated by subtracting the colony intensity with the background intensity. Raw CDE data, calculations, and scoring of the 4686 deletion mutants during light exposure is found in Additional file 3.

As the cell concentration at day 0 influences the cells’ growth response during illumination, and because the cell concentration at day 0 varied between the pinned colonies (Fig. 1f), both the CDE at day 0 and 6 were used in the scoring of light sensitivity. The settings of thresholds for identifying light-sensitive mutants were based on the experimental distribution of the 76 controls (*his3∆*; will be treated as wild-type controls) per plate (in total 1066 controls). The light-dependent growth of the controls distributed in a binary fashion, with control colonies with a CDE day 0 ≥ 121 CDE showing good growth under illumination while colonies with a CDE day 0 of < 121 exhibited variable scores, with control colonies with CDE day 0 ≤ 60 all being light-sensitive (Fig. 1h). Thus, two different criteria were used to select for light sensitive mutant strains: (i) a high confidence set: CDE_day0_ ≥ 121, and CDE_day6_ < 900 and (ii) a moderate confidence set: 61 ≤ CDE_day0_ ≤ 120, and CDE_day6_ < 400. The estimated rate of false positives, based on the distribution of the 1066 controls (*his3∆*), is 0.1% and about 5% for the high and moderate confidence set, respectively (Additional file 2: Table S1).

To avoid erroneously scoring generally slow-growing deletion strains as light-sensitive, we compared the growth of each mutant under light exposure to its corresponding growth in the dark. Because growth is much slower under light exposure, we compared the growth up until day 6 during light exposure to growth up until day 1 without light exposure. Finally, we normalized the light sensitivity of each deletion strain to the light sensitivity of the median of the 384 internal controls (*his3∆*) present on the same plate to estimate a double-normalized light growth (normalized to dark growth and to control strain light/dark growth), LGnorm_j_, for each deletion strain *j* as:

1 $$ {\mathrm{LG}\mathrm{norm}}_j=\frac{{\mathrm{LG}}_j/{\mathrm{DG}}_j}{\overset{\sim }{\mathrm{LG}\mathrm{ctr}/\mathrm{DGctr}}} $$

where LG_j_ and LG_ctr_ are the CDE for deletion strain j and the CDE for controls (*n* = 384), respectively, after 6 days of light growth, and DG_j_ and DG_ctr_ are the CDE for deletion strain j and CDE for controls (*n* = 384), respectively, after 1 day of dark growth. The median of the light/dark growth of the controls on each plate was used, and this was done independently for the high-confidence range and the medium-confidence range of initial cell densities (due to slightly different median values for the two ranges; in case of missing values for any of the ranges median values from the neighboring plates were used). We called light sensitive deletion strains in both the primary genome-wide screen and the confirmation assays as those with LGnorm_j_ < 0.75. Very slow growing deletion strains with less than a 1.5-fold increase in colony size during the first day of dark growth were excluded from the analysis.

For detecting resistant mutants with better growth than the control during illumination, we identified mutants with a CDE_day6_ > 1642 (52 mutants) using the same threshold criteria for cell density after pinning, 61 ≤ CDE_day0_, as for the light-sensitive mutants. The analytical window is much tighter for light resistance, and based on the growth of the control, the estimated rate of false positives is in this case 20%.

### The first confirmation assay—multiple pinning at various cell densities

For the follow-up confirmation test of the selected light-sensitive strains from the primary genome-wide screen, both haploid and diploid gene deletions were examined for 90 of the scored strains in the primary screen. To get better precision in our scoring in the confirmation test, each strain was serially pinned to four positions obtaining a concentration gradation of cell-densities (Additional file 1: Fig. S2). For the confirmation tests, the same pinning type we used as described above for the primary screen, but in a 1 × 96 to 1 × 384 format resulting in four colonies for each strain on a plate. In addition, we introduced a time-delay between each of the four pinnings, which due to cell sedimentation lead to lower amounts of cells being transferred for each pinning round. The following program options were used in the first confirmation assay for the liquid-to-agar transfer: 2D mixing for 10 repetitions at a distance of 0.1 mm from the bottom of the wells with a mixing speed of 25 mm/s and a mixing diameter of 1 mm. Light-sensitive mutants were scored as for the primary screen, with use of the experimental distribution of the internal control strains to define scoring thresholds as above—haploid set: high confidence CDE_day0_ ≥ 13 and CDE_day6_ < 900 (no moderate set defined since all control strains, even at low initial cell densities, grew well during illumination); diploid set: high confidence CDE_day0_ ≥ 105 and CDE_day6_ < 900 and moderate confidence 45 ≤ CDE_day0_ ≤ 104 and CDE_day6_ < 500. The control plates (no illumination) were wrapped up in aluminum foil and kept in the dark at 30 °C and were used to construct dark-normalized light-sensitivities of all mutants as above, using the same 75% threshold in relation to the control as for the primary screen. For example images from the time points used in the scoring of light sensitivity in dark and light, see Additional file 1: Fig. S3. Raw CDE data and the relevant calculations on the selected mutants and the controls in confirmation assay #1 is found in Additional file 4.

### The second confirmation assay—quantitative serial dilution drop test

For a subset of strains a second confirmation test based on quantitative serial dilution was performed, to obtain a quantitative measure of light sensitivity that would rank the mutants’ rate of growth during illumination (growth during illumination relative to their growth in the dark control). In this test, five tenfold serial dilutions with a starting dilution of OD_610_ ~ 1 (24 h of growth in liquid medium resulted in OD ≈ 4) were prepared and 7 μl of each dilution was spotted onto SC agar plates.

The serial dilution drop test was performed with the similar experimental set-up for illumination as in Fig. 1b, however, here with a distance of 33 cm to the light source. The distance to the lamp was thus slightly greater to lower the light stress since the cell density in this second confirmation assay was lower. The control plates (no illumination) were wrapped up in aluminum foil and kept in the dark at 30 °C. To obtain quantitative growth data the plates were scanned after 1, 2, 3, 5, and 6 days with a photo scanner (Epson Perfection V700) set in transmission mode. The plate scans from day 3 were used for quantitation by QuantityOne (Analysis Software version 4.6.8). To optimize the growth resolution and to get the best quantitative data, the second dilution of the dark control (OD_610_ ~ 0.1) was compared to the first dilution (OD_610_ ~ 1) of the light stressed cells, since there are different growth rates in light and dark. We here also compensated for slower growth in the dark of the deletion strains compared to wild type, by applying a relative cell-spot intensity per area unit after background subtraction (INT/mm^2^) to calculate the strain specific light/dark ratio. For a number of slow growing strains, the light value was extracted at day 5 (same dilutions) for a more reliable quantification. Statistical significance was assessed for each strain in relation to the control by two-sided Student *t* tests assuming equal variance.

### Functional enrichment using the Gene Ontology

Enrichments of specific cellular functions among light-sensitive mutants were determined using SGD yeast GO slim process ontology, comparing the frequency of each cellular process among light-sensitive deletion mutants. Mutants corresponding to dubious genes were excluded and processes encoded by < 25 genes were not evaluated. Significance of deviations between the light-sensitive mutants and the reference set (all mutants for which a comparable measure of growth at the start, CDE_day0_ at least 61) was determined using a hypergeometric distribution and correcting for multiple hypotheses using a false discovery rate (FDR, *q* = 0.06).

GO enrichments among light-resistant mutants were determined using the SGD yeast GO slim components ontology.

### Fluorescence microscopy analysis of Msn2-GFP nuclear localization

Images were acquired using an automated epi-fluorescence microscope (TE2000E-PFS, Nikon Instruments) equipped with a 60X oil immersion objective (NA1.4, Plan Apochromatic, Nikon Instruments) and an electron-multiplying CCD camera (iXon DU-885-CS0-#VP, Andor Technology). Green fluorescent protein (GFP) excitation of Msn2-GFP and simultaneous stress induction were performed as previously described [44]. In short, the cells were illuminated with continuous blue light (470 ± 20 nm) from a standard mercury lamp with a constant intensity of 82 mW/cm^2^ (820 W/m^2^). Images were captured continuously for 60 min in one focal plane with an acquisition time of 4 s, i.e., the temporal resolution corresponds to roughly 4 s, using the software NIS-elements (Nikon). The cells were kept in a FCS2 perfusion chamber (Bioptechs Inc.) at 28 °C and immobilized using concanavalin A coated coverslips (0.1 μg/μl in 0.01 M PBS for 1 h). After each experiment, cells surrounding the illuminated area were imaged in order to ensure that only cells that had been illuminated exhibited nuclear Msn2 localization, thus excluding possible errors in the setup or uncontrollable factors that could lead to stress induction.

Image and signal analysis was performed as previously described in [44] by using the software CellStat (Fraunhofer-Chalmers Centre for Applied Mathematics, CellStat, Gothenburg), which identifies cell contours and extracts fluorescent data [79].

Fourteen strains from those that were identified as hits in the second confirmation assay (Fig. 2) were selected to investigate the Msn2 nucleocytoplasmic response. They were, together with the wild type strain, transformed with a plasmid [80] based on YCplac111 (*LEU2* marker), containing an Msn2-GFP fusion protein controlled by the *ADH1* promoter. Strains were grown at 30 °C in synthetic defined medium (as described above) except that complete supplement mixture was without leucine to stabilize the plasmid. Overnight cultures were inoculated in the morning and grown to an OD_600_ of 0.4–0.5 at the start of the microscopy experiment. A reference experiment with the wild type strain was run each experimental day to eliminate any systematic artifacts. These control strains were also used as a way of classifying the mutants according to their nucleocytoplasmic shuttling of Msn2.

### Construction of mutants for opsin-like genes

The yeast *S*. *cerevisiae* carries three genes that encode opsin-like proteins, *MRH1*, *YRO2*, and *HSP30*. Diploid mutant strains were constructed by mating haploid strains of opposite mating types of each of the opsin-like single mutants (i.e., *mrh1∆*, *yro2∆*, and *hsp30∆*). Haploid cells were taken from freshly growing colonies and mixed in 50 μl YPD medium to produce diploid strains of different double-mutant combinations. Diploid strains were then transferred to 1% potassium acetate (KAC) plate for sporulation and were incubated at 30 °C for 2 to 3 days. The ascal wall was digested with the enzyme lyticase and the reaction was stopped by using ice-cold sterile MilliQ water. The separation of the four ascospores from individual asci was done by micromanipulation (the Singer MSM series 200 System was used). To obtain the *mrh1∆yro2∆hsp30∆* triple mutant, the haploid *mrh1∆* was mated with the double mutant *yro2∆hsp30∆*, and the obtained diploid sporulated and individual spores examined as described above. The correctness of all mutants was confirmed by nested PCR for each of the opsin-like genes and electrophoretic analysis of the obtained DNA fragments.

Different combinations of diploid mutants and triple mutants were produced and further tested for their light sensitivity by following the quantitative serial dilution drop test as described above but this time using the Scan-o-matic software for quantification of cell density [81]. Instead of BY4743 strain, the BY4742 strain was used as a reference strain and *hog1Δ* was used as a light-sensitive control. Statistical significance (alfa < 0.05) was assessed for each strain in relation to the wt control by two-sided Student *t* tests assuming equal variance. There were slightly different numbers of independent experiments for the different mutants: BY4742/wt, *yro2Δmrh1Δ* and triple deletion mutants *n* = 6, *hog1Δ*, *hsp30Δ* and *hsp30Δmrh1Δ n* = 5, *mrh1Δ n* = 4, while *n* = 3 in the case of *yro2Δ* and *hsp30Δyro2Δ*.

### *In silico* predicted absorption spectra of diphthamide intermediates

In silico prediction of absorption spectra of diphthamide intermediates were performed in line with earlier theoretical work on the molecular dynamics of EF2 and its His699 modification into diphthamide [36]. The structures of eEF2 from *Saccharomyces cerevisiae*, alone and in complex with sordarin (interacts with and inhibits eEF2), were solved at 2.9 and 2.1 Å resolution, respectively [82]. The composition of the four systems studied herein (HIS, ACP, DTI, DTA) is described in [36] and contains roughly 300,000 atoms. In each case, the protein was first centered in a periodic box with the box size adapted to extend 1.0 nm from the protein edges. Water molecules were added and the counterion concentration modified to have a charge neutral system using the genion algorithm of Gromacs.

The system was subjected to equilibration at *T* = 300 K and *P* = 1 bar, and a trajectory of 50 ns was run at the same conditions. The time step was set to 2 fs, using the Lincs algorithm to constrain all bonds [83], and the leap-frog algorithm for integration [84]. Electrostatic forces were treated using particle-mesh Ewald (PME) summation and the cutoff was 10 Å both for electrostatics and van der Waals interactions. The simulation was performed with a velocity rescaling thermostat with a 0.1 ps coupling constant, and a Parrinello-Rahman barostat (NPT ensemble) with a 2.0 ps coupling constant. The Amber ff99sb [85] force field was used throughout all molecular dynamics simulations, containing updated backbone ϕ and ψ torsions and improved sidechain torsion angles. Including a modified amino acid in the system requires the calculation of new parameters and constants to be included in the force field. Determination of missing parameters in the ff99sb force field was performed through optimization of the particular system to a minimum using ground state Hartree-Fock calculations with a 6-31G(d) basis set. It was then possible to use Antechamber, to extract the data from the output file and converting them into the Amber ff99 force field parameters required in Gromacs. All ab initio calculations reported herein were carried out employing Gaussian 09 [86].

Time-dependent density functional theory (TD-DFT [87]) is an extension of density functional theory (DFT [88]). The selection of the particular functional to use is an important task and has been studied thoroughly. Several DFT functionals correctly predict the excitation energies in the wavelength range from 300 to 600 nm, such as ωB97XD [89], B3LYP [90], PBE0 [91], and M06 [92]. Herein, ωB97XD was the selected functional employed in all DFT calculations. The coordinates of all the atoms constituting eEF2 were extracted every 5 ns of the MD simulation and stored in separate files to identify relevant atomic arrangements. This was done for each derivative of His699 (HIS, ACP, DTI, and DTA). In order to perform the TD-DFT calculations (to obtain molecular orbitals and absorption spectra) within a reasonable time, only the atomic coordinates of the three residues of interest (His583, His694, and (modified) His699; Additional file 1: Fig. S4) were considered from each snapshot. In these calculations, the local environment (i.e., other nearby amino acid residues or solvent molecules) was not included. Since the molecular orbitals (and therefore the corresponding spectra) are highly dependent on the atomic arrangement, it is important to consider the distinct conformations found, e.g., the two states for the ACP modification of eEF2. Therefore, 10 snapshots were selected along the trajectories (Additional file 1: Fig. S5), and TD-DFT calculations were performed on each of them. Molecular orbitals and thereafter absorption spectra were then computed directly from the coordinates extracted from the MD simulations without any further optimization.

The graphs show that the modified His-residues suffer fluctuations, thus describing several possible atomic arrangements during the MD simulation. In particular, the root-mean-square deviation (RMSD) of ACP699 and DTI699 suggest the existence of two stable states that the residues oscillate between. One could envisage that this is also the case for DTA699; however, the major fluctuations occur during the equilibration stage, and the atoms in this modification remain very stable during the production phase. His699 of the unmodified eEF2 show only a slight variation relating to one single state during the entire simulation trajectory, indicating that the two intermediates ACP and DTI have more complex interactions with their surroundings.

The cumulated absorptions for the different snapshots are shown in Additional file 1: Fig. S6 pointing out that none of the intermediates (HIS, ACP, DTI, or DTA) can absorb visible light through the HOMO→LUMO electronic transition. The lowest-energy transitions (longest wavelengths) occur around 340–350 nm for all four systems studied, with more pronounced transitions noted for ACP.

### Glycogen assay

The accumulation of glycogen was assayed as a reporter for altered PKA activity (high PKA leads to low levels of glycogen [45]) by exposing cells, grown for 3 days from inoculums consisting of 10 μl drops of mid-exponential phase (OD_600_ 0.5) cultures diluted 10-fold, to iodine vapor in petri-dishes as earlier described [54].

### The FRET-based PKA sensor AKAR4

The FRET-based PKA sensor AKAR4 was subcloned from plasmid pcDNA3-AKAR4 [93] into plasmid pRS416-GPD [94] using restriction enzymes BamHI and EcoRI. The resulting plasmid pRS416-GPD-AKAR4 was sequence verified prior to use. Detection of cyan fluorescent protein CFP➔ yellow fluorescent protein YFP FRET in the AKAR4 sensor was performed as described previously [47]. CFP was excited at 427/10 nm, YFP was excited at 504/6 nm, and emission was monitored using a Semrock dual bandpass filter (part no: FF01-464/547). For unstressed cells, a light intensity of 26 μW across the illuminated area (2.47 × 10^−4^ cm^2^; giving a light intensity in the order of 1000 W/m^2^) was used since imaging at this light-intensity did not increase Msn2 nuclear localization for 60 min following the start of illumination [14]. Images were acquired using an automated epi-fluorescence microscope (Olympus IX81) equipped with a × 60 oil immersion objective (numerical aperture 1.4, PlanApoN × 60/1.42 Oil, Olympus) and an electron-multiplying charge-coupled device camera (12-bit Hamamatsu camera). The yeast cells were kept in a heated perfusion chamber (FCS2, Bioptechs Inc.) at 28 °C to avoid heat-induced stress responses. The objective was heated to 26.2 °C (according to the manufacturer’s instructions) to maintain a stable temperature in the perfusion chamber. The cover glasses were precoated for 1.5 h with protein concanavalin A, 0.5 μg μl^−1^ in 0.01 M PBS, to immobilize yeast cells on the surface.

## Supplementary information

**Additional file 1: Figure S1.** Example images in dark and light from the genome-wide screen from one of the plates (plate 1) from the time-points used in the scoring of light-sensitivity. **Figure S2.** Design and results of the first confirmation assay for the selected deletion mutants in the diploid BY4743 background. A) Each strain was pinned into four different positions on the plate (like a quadrant) with the order of pinning indicated for one of the strains in the upper left corner. A slight delay between the four pinnings was imposed, resulting in a dilution series. B) The quantification of the overall dilution between the repeated pinnings summed over all the tested 96 strains. The data shown represents box plots, with the line in the box indicating the median and box boundaries indicating 25 and 75 percentiles. C) Light-sensitivity of the mutants selected from the initial genome-wide screen that were also scored as light-sensitive in the first confirmation assay, only the hits based on criterium I are shown. The green dots indicate light-sensitive hits and the black dots indicate no light-sensitivity (no hits) according to our definition based on the experimental distribution of the included control strains. The x-axis shows the various initial cell densities (CDE) at day 0. The mutants are sorted according to their light-sensitivity, where a light-sensitive hit with a high CDE value at day 0 is regarded a stronger hit than a strain with a lower start cell density intensity. Note however that the range of starting cell densities differs between the mutants (a consequence of the automated pinning) and therefore the sorting only reflects a tentative strength of a hit. **Figure S3.** Images from different time-points in dark and light used in the scoring of light-sensitivity from confirmation assay #1 for the selected mutants of the haploid collection. Indicated on the images are the gene-deletions analyzed. Each mutant is represented by four colonies/dots, with slightly different initial cell-numbers as a result of our modified pinning regime – see Materials and Methods and Figure S2 for more details. **Figure S4.** The structure of eEF2 in *S*. *cerevisiae*, based on PDB id 1N0V. The three histidine residues (His583, His694 and His699) discussed in the text are displayed in ball-and-stick representation to the far left in the structure and in the zoom-in representation. **Figure S5.** The root-mean-square deviation (RMSD) of all atoms of residue His699 in yeast EF2. Black crosses represent the snapshots used for the UV-visible spectral analysis (Fig. S6). **Figure S6.** Absorption wavelengths from each snapshot of the four eEF2 derivatives.

**Additional file 2: Table S1.** Summary statistics for the light-stress genome-wide screen of the haploid strain BY4741 and for the subsequent first confirmation assay.

**Additional file 3. **Raw CDE data on all 4686 deletion mutants and the 1066 controls (*his3∆*) for day 0 and day 6 during light exposure and for day 0 and day 1 in the dark. The file includes the following datasheets: Sheet 1. “read me”, contains some general information about the data presented. Sheet 2: “mutants_haploid collection”, contains the raw data and the calculations that are part of our scoring regime for deletion mutants (the haploid collection used for the genome-wiede screen). Sheet 3: “controls (*his3∆*)”, contains the raw data and the calculations that are part of our scoring regime for the control (*his3∆*) indicating the plate-wise median dark-normalized light-growth for each confidence range used for the normalization of the mutants on the same plate.

**Additional file 4.** Raw CDE data on the selected mutants for the confirmation assay #1 and there corresponding controls for day 0 and day 6 during light exposure and for day 0 and day 1 in the dark. The file includes the following datasheets: Sheet 1. “read me”, contains some general information about the data presented. Sheet 2: “ confirmation asay 1 - haploids”, contains the raw data and the calculations that are part of our scoring regime for the selected haploids. Sheet 3: “control strains_- haploids”: contains the raw data and the calculations that are part of our scoring regime for the control (his3∆) for the haploids indicating the median dark-normalized light-growth for the high-confidence range used for the normalization of the mutants (all control strains grew well even at low initial cell-densities and thus all represented the high-confidence range). Sheet 4: “ confirmation asay 1 - diploids”, contains the raw data and the calculations that are part of our scoring regime for the selected diploids. Sheet 5: “control strains - diploids”, contains the raw data and the calculations that are part of our scoring regime for the control (wild type, BY4743) indicating the median dark-normalized light-growth for each confidence range used for the normalization of the mutants.

**Additional file 5. **Individual values for figures where the number of independent replicates is less than 6 (*n* < 6). Each sheet in the Excel-file is named by the figure the data represent.

## Data Availability

Data generated and analyzed during this study are included in the published article and its supplementary information files. Additional files 3 and 4 contain all the raw data and the corresponding calculations for the primary screen of light sensitivity for the 4686 deletion mutants and the confirmation assay #1, respectively. Additional file 5 provides individual values for figures where the number of independent replicates is less than 6 (*n* < 6).
