# Supplementary material for: Protein kinase A controls yeast growth in visible light
Source: BMC Biol. 2020 Nov 16;18:168. doi: 10.1186/s12915-020-00867-4 (PMC7667738; doi:10.1186/s12915-020-00867-4)
Supplement: Supplementary file 1 — Additional file 1: Figure S1. Example images in dark and light from the genome-wide screen from one of the plates (plate 1) from the time-points used in the scoring of light-sensitivity. Figure S2. Design and results of the first confirmation assay for the selected deletion mutants in the diploid BY4743 background. A) Each strain was pinned into four different positions on the plate (like a quadrant) with the order of pinning indicated for one of the strains in the upper left corner. A slight delay between the four pinnings was imposed, resulting in a dilution series. B) The quantification of the overall dilution between the repeated pinnings summed over all the tested 96 strains. The data shown represents box plots, with the line in the box indicating the median and box boundaries indicating 25 and 75 percentiles. C) Light-sensitivity of the mutants selected from the initial genome-wide screen that were also scored as light-sensitive in the first confirmation assay, only the hits based on criterium I are shown. The green dots indicate light-sensitive hits and the black dots indicate no light-sensitivity (no hits) according to our definition based on the experimental distribution of the included control strains. The x-axis shows the various initial cell densities (CDE) at day 0. The mutants are sorted according to their light-sensitivity, where a light-sensitive hit with a high CDE value at day 0 is regarded a stronger hit than a strain with a lower start cell density intensity. Note however that the range of starting cell densities differs between the mutants (a consequence of the automated pinning) and therefore the sorting only reflects a tentative strength of a hit. Figure S3. Images from different time-points in dark and light used in the scoring of light-sensitivity from confirmation assay #1 for the selected mutants of the haploid collection. Indicated on the images are the gene-deletions analyzed. Each mutant is represented by four colonies/dots, with slight [file 12915_2020_867_MOESM1_ESM.pdf]

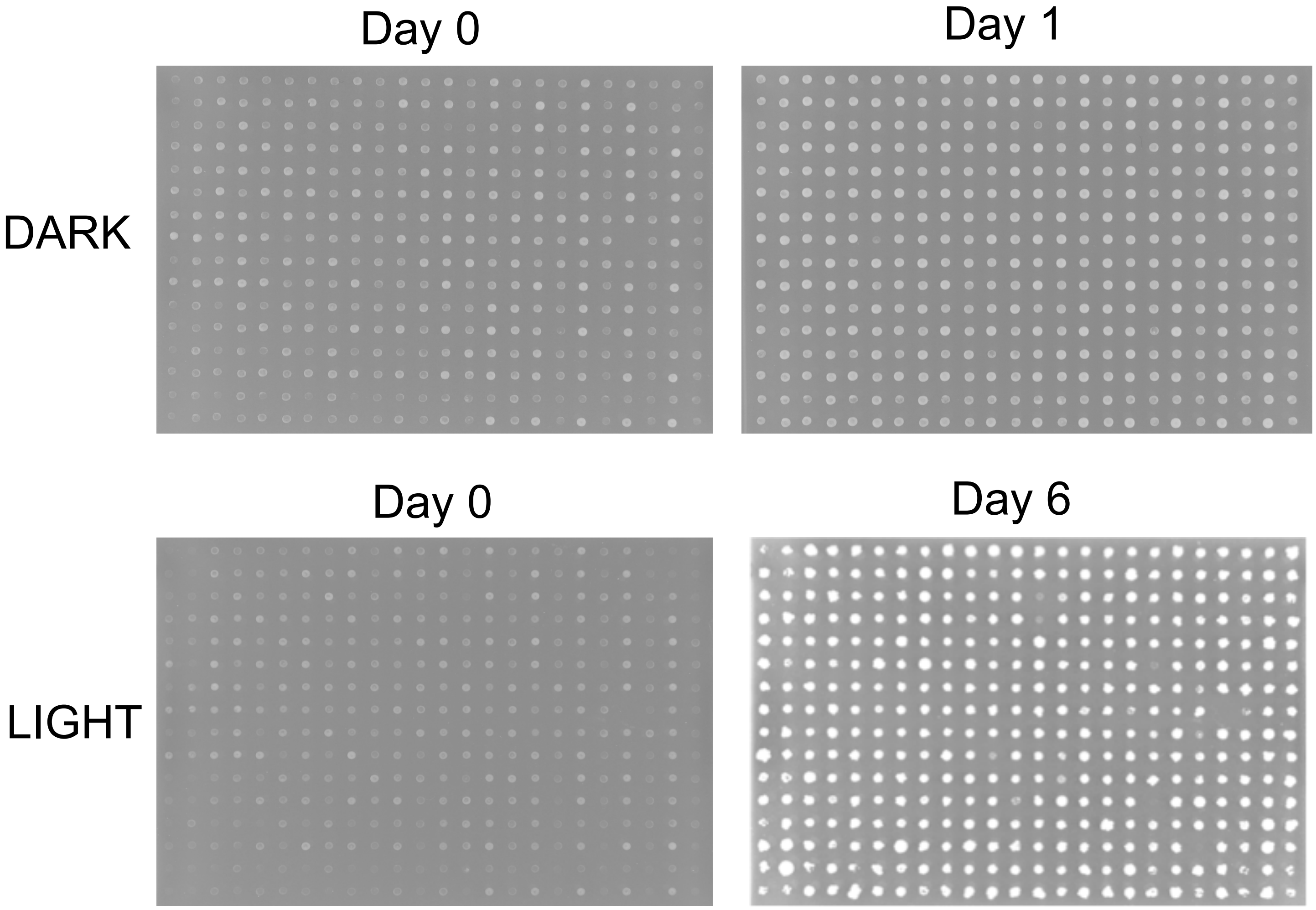

Figure S1: Example images in dark and light from the genome-wide screen from one of the plates (plate 1) from the time-points used in the scoring of light-sensitivity.

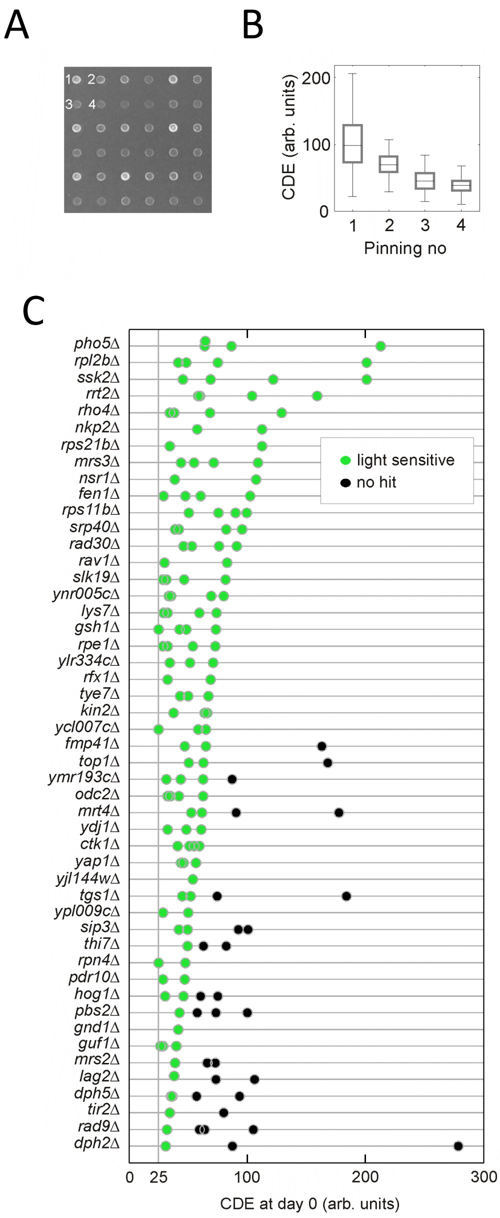

Figure S2. Design and results of the first confirmation assay for the selected deletion mutants in the diploid BY4743 background. A) Each strain was pinned into four different positions on the plate (like a quadrant) with the order of pinning indicated for one of the strains in the upper left corner. A slight delay between the four pinnings was imposed, resulting in a dilution series. B) The quantification of the overall dilution between the repeated pinnings summed over all the tested 96 strains. The data shown represents box plots, with the line in the box indicating the median and box boundaries indicating 25 and 75 percentiles. C) Light-sensitivity of the mutants selected from the initial genome-wide screen that were also scored as light-sensitive in the first confirmation assay, only the hits based on criterium I are shown. The green dots indicate light-sensitive hits and the black dots indicate no light-sensitivity (no hits) according to our definition based on the experimental distribution of the included control strains. The x-axis shows the various initial cell densities (CDE) at day 0. The mutants are sorted according to their light-sensitivity, where a light-sensitive hit with a high CDE value at day 0 is regarded a stronger hit than a strain with a lower start cell density intensity. Note however that the range of starting cell densities differs between the mutants (a consequence of the automated pinning) and therefore the sorting only reflects a tentative strength of a hit.

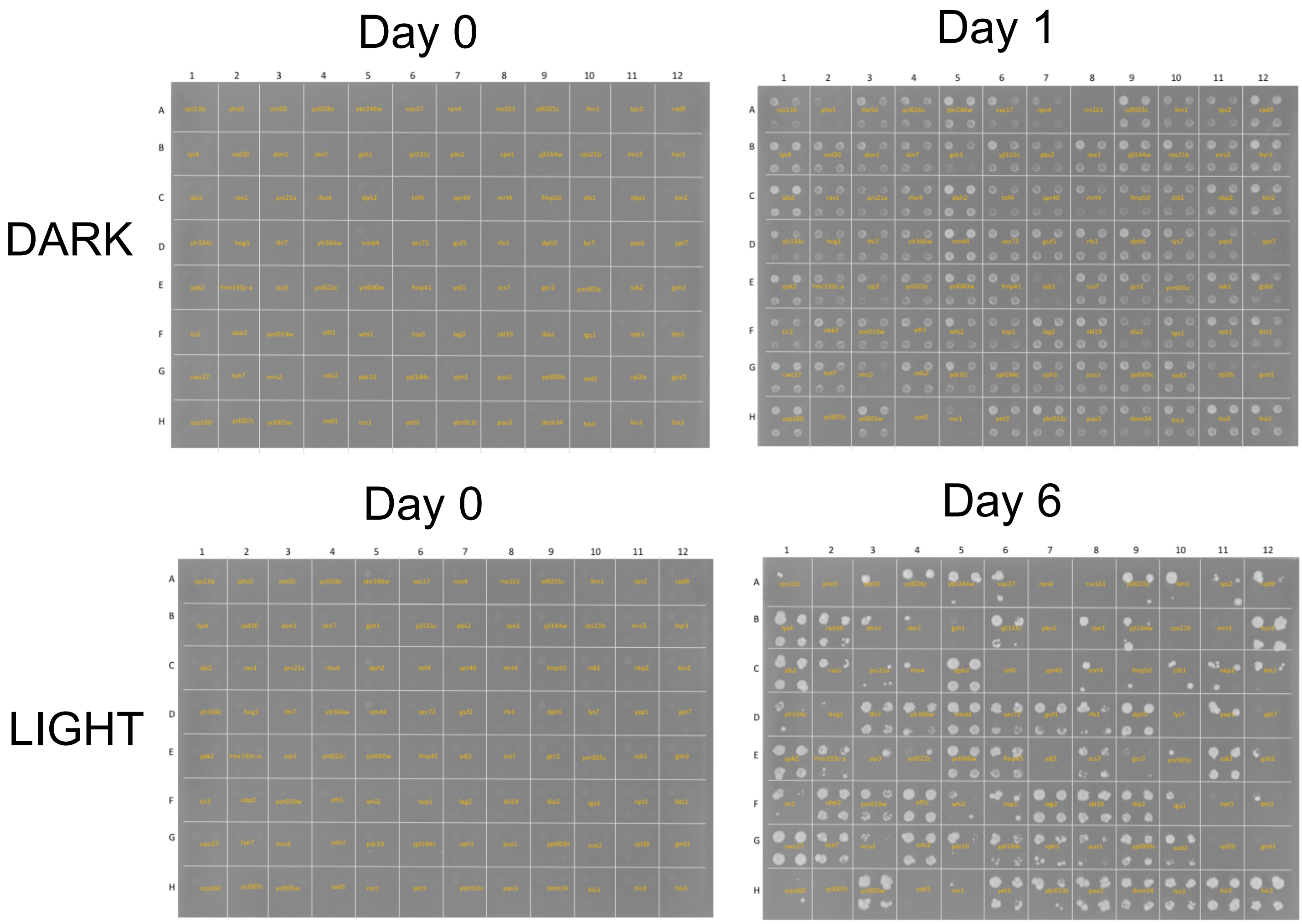

Figure S3: Images from different time-points in dark and light used in the scoring of light-sensitivity from confirmation assay #1 for the selected mutants of the haploid collection. Indicated on the images are the the gene-deletions analysed. Each mutant is represented by four colonies/dots, with slightly different initial cell-numbers as a result of our modified pinning regime – see Materials and Methods and figure S2 for more details.

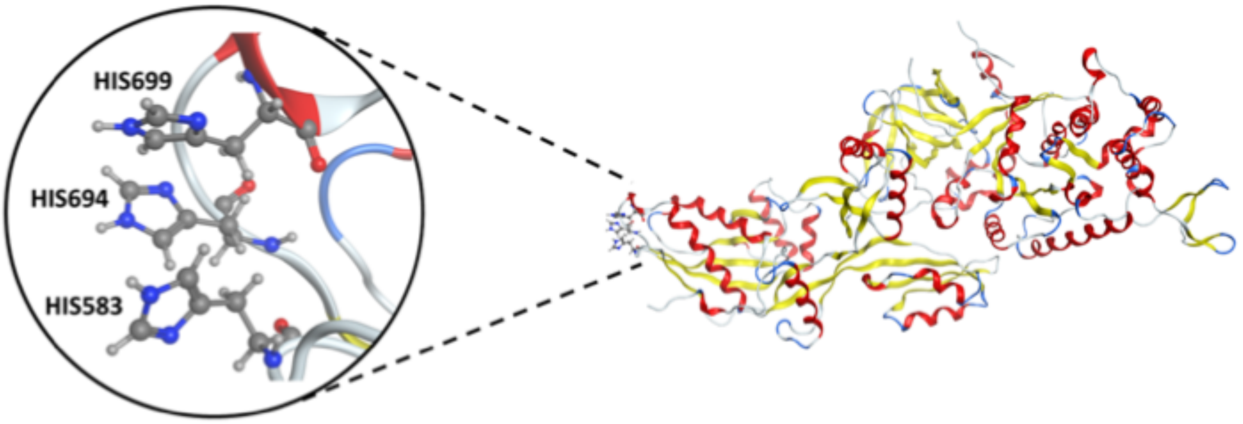

Figure S4. The structure of eEF2 in S. cerevisiae, based on PDB id 1N0V. The three histidine residues (His583, His694 and His699) discussed in the text are displayed in ball-and-stick representation to the far left in the structure and in the zoom-in representation.

Molin M et al Figure S4

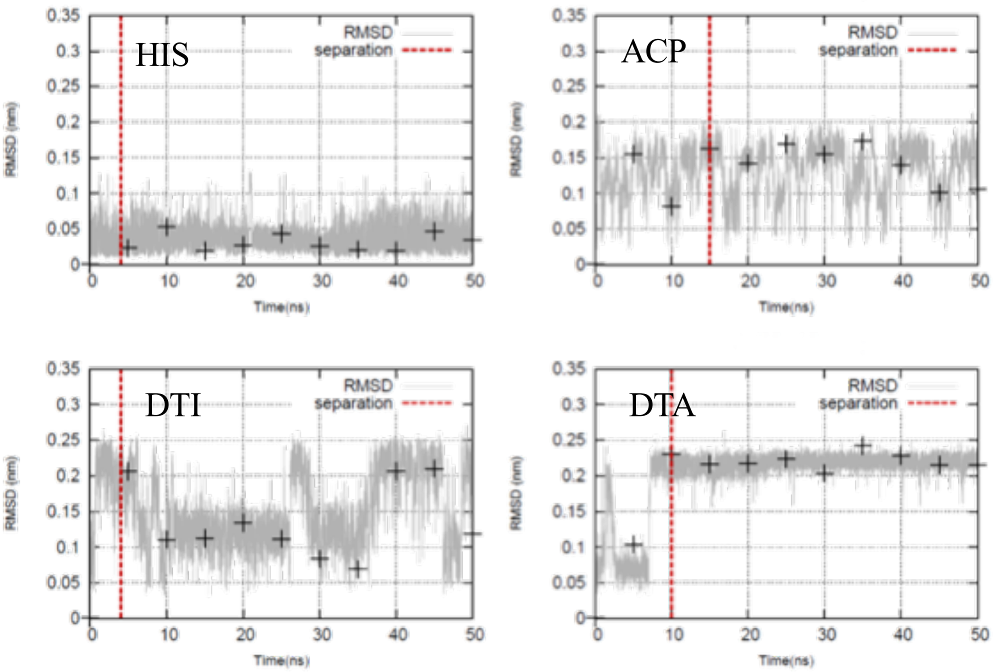

Figure S5. The root-mean-square deviation (RMSD) of all atoms of residue His699 in yeast EF2.

Molin M et al Figure S5

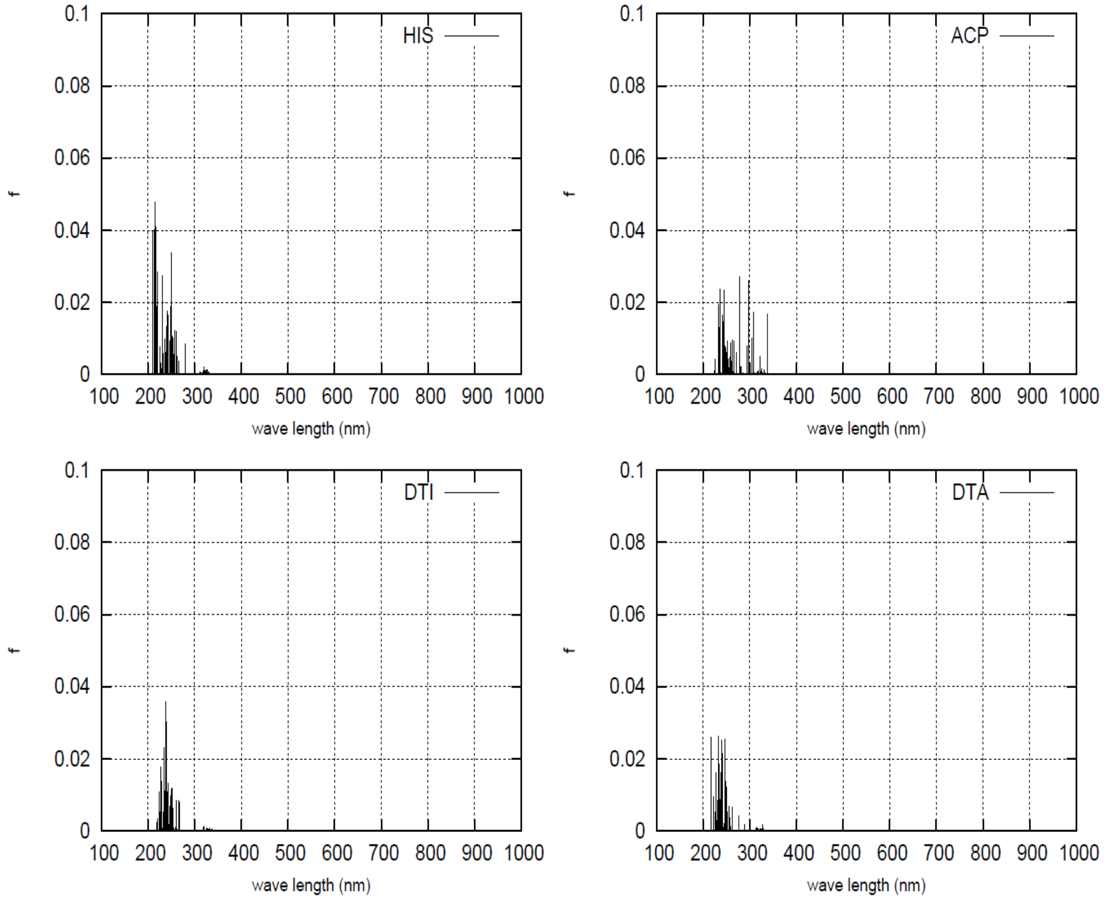

Figure S6. Absorption wavelengths from each snapshot of the four eEF2 derivatives.

Molin M et al Figure S6
