# Supplementary material for: Protein kinase A controls yeast growth in visible light
Source: BMC Biol. 2020 Nov 16;18:168. doi: 10.1186/s12915-020-00867-4 (PMC7667738; doi:10.1186/s12915-020-00867-4)
Supplement: Supplementary file 2 — Additional file 2: Table S1. Summary statistics for the light-stress genome-wide screen of the haploid strain BY4741 and for the subsequent first confirmation assay. [file 12915_2020_867_MOESM2_ESM.pdf]

Table S1: Summary statistics for the light-stress genome-wide screen of the haploid BY4741 deletion mutant collection and for the subsequent first confirmation assay. The two selection criteria – criteria I: "high confidence" (CDE light day0 > 121; light day6 < 900) and "medium confidence" (CDE 60 < light day0 < 121; light day6 < 400), and criteria II: specific light-sensitivity growth measure (normalized to the mutants' growth in the dark) that is normalized to the control strains (n=384) on each plate– and their respective outcomes in false positives are indicated. The number of false positives for the various selection criteria are based on the outcome of the 1,066 control strains. Indicated are also the total number of mutant colonies in the respective confidence-ranges. The numbers in the table relating to the genome-wide collection refers to the total number of mutant colonies screened (4,858), which corresponds to 4,697 unique mutant strains: 205 "high-confidence" mutant colonies corresponds to 205 unique mutants and 276 "medium-confidence" mutant colonies corresponds to 267 unique mutants (the latter are the numbers given in the discussions in the main text).

	**False positive**<br><br>**Based on control strain in primary screen**		**False positives**<br><br>**Based on re-tested selected light-sensitive mutants in confirmation assay #1**		**Light-sensitive mutant colonies**		
**Selection criteria for hits**	**Number/ total**	**%**	**Number/ total**	**%**	**Number of mutants in that range**	**Criterium I**	**Criterium I and II**
**High confidence**<br><br>Light:day0≥121; day6<900	0/1,066	0	3/22	14	1,591	227	205
**Moderate confidence**<br><br>Light:60<day0<121; day6<400	50/1,066	4.7	16/52	31	2,026	276	276
